# Neuroanatomy-Informed Brain–Machine Hybrid Intelligence for Robust Acoustic Target Detection

**DOI:** 10.34133/cbsystems.0438

**Published:** 2025-10-17

**Authors:** Jianting Shi, Jiaqi Wang, Weijie Fei, Aberham Genetu Feleke, Luzheng Bi

**Affiliations:** School of Mechanical Engineering, Beijing Institute of Technology, Beijing 100081, China.

## Abstract

Sound target detection (STD) plays a critical role in modern acoustic sensing systems. However, existing automated STD methods show poor robustness and limited generalization, especially under low signal-to-noise ratio (SNR) conditions or when processing previously unencountered sound categories. To overcome these limitations, we first propose a brain–computer interface (BCI)-based STD method that utilizes neural responses to auditory stimuli. Our approach features the Triple-Region Spatiotemporal Dynamics Attention Network (Tri-SDANet), an electroencephalogram (EEG) decoding model incorporating neuroanatomical priors derived from EEG source analysis to enhance decoding accuracy and provide interpretability in complex auditory scenes. Recognizing the inherent limitations of stand-alone BCI systems (notably their high false alarm rates), we further develop an adaptive confidence-based brain–machine fusion strategy that intelligently combines decisions from both the BCI and conventional acoustic detection models. This hybrid approach effectively merges the complementary strengths of neural perception and acoustic feature learning. We validate the proposed method through experiments with 16 participants. Experimental results demonstrate that the Tri-SDANet achieves state-of-the-art performance in neural decoding under complex acoustic conditions. Moreover, the hybrid system maintains reliable detection performance at low SNR levels while exhibiting remarkable generalization to unseen target classes. In addition, source-level EEG analysis reveals distinct brain activation patterns associated with target perception, offering neuroscientific validation for our model design. This work pioneers a neuro-acoustic fusion paradigm for robust STD, offering a generalizable solution for real-world applications through the integration of noninvasive neural signals with artificial intelligence.

## Introduction

Sound target detection (STD) is a reconnaissance method that uses acoustic sensors to detect the presence of targets in the environment. Sound sensors have the advantages of low cost and easy maintenance. Acoustic signals are not easily blocked compared with optical and radar reflection signals, making STD a broad application prospect in security protection, environmental reconnaissance, and other fields. It has attracted plenty of attention from the academic community in recent years.

Current research focuses on STD as a classification problem in machine learning, and many related works have proposed STD methods and systems. For example, Lopatka and Czyzewski [1] proposed a parallel sound data stream processing method for sound detection of dangerous events (such as screaming and gunfire). The marked difference from previous studies is that this method does not extract features after buffering sound events as a whole. Instead, the time domain, frequency domain, and Mel frequency cepstral coefficients (MFCCs) of short-time sound data were extracted as classification features after the sound event appeared, and support vector machine (SVM) was used for continuous judgment. The results showed that this method significantly reduced the detection time, classification delay, and classification processing time of sound events compared with other methods. Waldekar and Saha [2] proposed a Mel-scaled feature based on wavelet transform for the detection of environmental sound events and used SVM with RBF kernel for classification. The results showed that this method had higher accuracy on multiple test datasets than traditional methods, such as MFCC+GMM (Gaussian mixture model). Aiming at the problem of how to determine the detection threshold in sound event detection, Kong et al. [3] proposed a convolutional neural network (CNN) classification algorithm with automatic threshold optimization and tested the performance of the algorithm on the Task 4 of Detection and Classification of Acoustic Scenes and Events (DCASE) 2017. The results showed that the classification model with automatic threshold optimization had a higher F1 score in marking and detecting sound events than the model without optimized threshold.

However, some studies demonstrated that the robustness of current STD methods is flawed. Current STD methods usually have good recognition efficiency and accuracy under the controlled conditions. However, in actual situations, key parameters (such as signal-to-noise ratio [SNR] and sound intensity of the target sound signal) change sharply due to changes of environmental factors and detection targets. In this case, such methods cannot perform well against the actual problem conditions. In 2005, Clavel et al. [4] pointed out that the reduction of SNR led to a sharp decline in detection performance. Under the premise of the same training strategy, when the SNR decreases from 20 to 5 dB, the detection accuracy decreases significantly. Papadimitriou et al. [5] also found this problem. The STD model was first trained using 30 dB SNR data and then tested using 30 dB and −5 dB SNR data. The performance of the model on the low SNR test set was significantly lower than that on the high SNR test set (accuracy decreased by 36.51% and recall rate decreased by 57.58%). Ren et al. [6] demonstrated the influence of SNR on detection performance in the study of dangerous sound detection In the same noise scenario, when the SNR was −15 dB, the detection error rate of the same acoustic target on the test set reached 37.38%, which was 30.2% higher than that when the SNR was −5 dB. Aiming at the problem of sound detection with low SNR, Li and Wu [7] mixed background noise and sound events to form noisy samples, extracted sub-band energy features, and then used random forest algorithm to classify them. The results showed that the method could maintain an average detection rate of 67.1% when the SNR is −5 dB. A subsequent paper in 2018 [8] showed that the scheme based on multi-band energy features and random forest classifiers performed better than traditional methods (such as MFCC+SVM) at low SNR (−10 dB). However, the recognition rate was still below 50%.

Thus, fully autonomous STD systems still face many challenges. Human involvement is still necessary in some cases [9,10]. In such cases, manual recognition accuracy is higher than machine learning algorithms. In the literature of neuroscience, psychology, and auditory science, a large number of studies have explored and proved the robustness and strong generalization of the human hearing system [11–14]. However, manual recognition has a slow processing speed. It is challenging to deal with massive data processing, making it difficult to meet the requirements of real-time STD systems.

Brain–computer interface (BCI) is a new human–computer interaction method that can operate external devices by directly “translating” brain activity. Certain studies have demonstrated the capability of BCIs in detecting image, video, or sound targets by decoding electroencephalogram (EEG) signals while humans are seeking the targets [15–17]. Our previous study has employed a traditional SVM algorithm as the EEG decoder to conduct a preliminary study, and has shown the feasibility of an auditory BCI-based STD system in complex acoustic scenes [18]. Experimental results showed that even when the SNR reduced to −10 dB, humans’ detection ability remained stable. However, most existing EEG decoding methods are either general purpose or designed for nonauditory task, with few algorithms specifically developed for target recognition in complex auditory scenes [19–21]. A more pressing issue is that the unsteady characteristics of EEG signals led to a high false alarm rate (FAR) when the BCI was used alone, and the BCI method still cannot solve the problem of human fatigue, which undoubtedly limits its effectiveness in practical applications.

To address the above limitations, in this paper, we first present a detailed source-level analysis of EEG data from offline experiments, revealing neural activation patterns associated with sound target perception in complex auditory scenes. The results provide neuroscientific insight into how the human brain processes auditory targets under noisy conditions. We propose a novel EEG decoding network, Triple-Region Spatiotemporal Dynamics Attention Network (Tri-SDANet), which integrates neuroanatomical priors derived from source analysis into a task-informed model architecture. Unlike conventional architectures that treat EEG channels uniformly, Tri-SDANet adopts a neuroanatomy-based spatial partitioning strategy. It divides EEG electrodes into 3 functionally distinct brain regions and models each region using dedicated spatiotemporal convolutional branches with biologically informed receptive fields. To further capture the temporal dynamics of auditory target detection, the model employs multi-scale temporal convolution and dynamic attention weighting, enabling the selective enhancement of neural signatures across early, middle, and late processing stages. Additionally, a temporal gating module adaptively modulates region-specific activations to emphasize task-relevant temporal segments. This design improves feature interpretability and task relevance, thereby significantly enhancing decoding accuracy in complex auditory scenarios. Compared with the state-of-the-art models on the dataset of BCI offline experiments, Tri-SDANet outperforms them on multiple evaluation metrics.

Furthermore, to mitigate the high FAR of stand-alone BCIs and the performance degradation of conventional STD systems in low-SNR and unfamiliar environments, we propose a confidence level-driven brain–machine hybrid intelligence framework. Unlike simple score-based fusion schemes, our approach introduces a principled confidence interval optimization strategy that adaptively integrates BCI outputs only when the automatic detector exhibits uncertainty. A multi-objective evaluation function is designed to balance correct detections and error reduction. This enables the system to selectively invoke neural decoding only when needed, reducing human workload while enhancing robustness and generalization. Extensive streaming-mode detection experiments demonstrate that this hybrid framework effectively leverages the strengths of both BCI and automatic detection methods while mitigating their respective weaknesses, achieving superior performance particularly in challenging low-SNR scenarios and with previously unseen target types.

In summary, the key contributions of this work are as follows:

1. This work pioneers a neuro-acoustic fusion paradigm for robust STD, offering a generalizable solution for real-world applications.
2. We design a neuroanatomically guided decoding model, Tri-SDANet, which achieves state-of-the-art EEG decoding performance for auditory target detection.
3. We propose an adaptive confidence-aware brain–machine hybrid strategy that effectively integrates BCI and automatic STD algorithms to improve detection robustness and generalization under complex acoustic conditions.

## Materials and Methods

### Paradigm and data acquisition

**Fig. 1.**
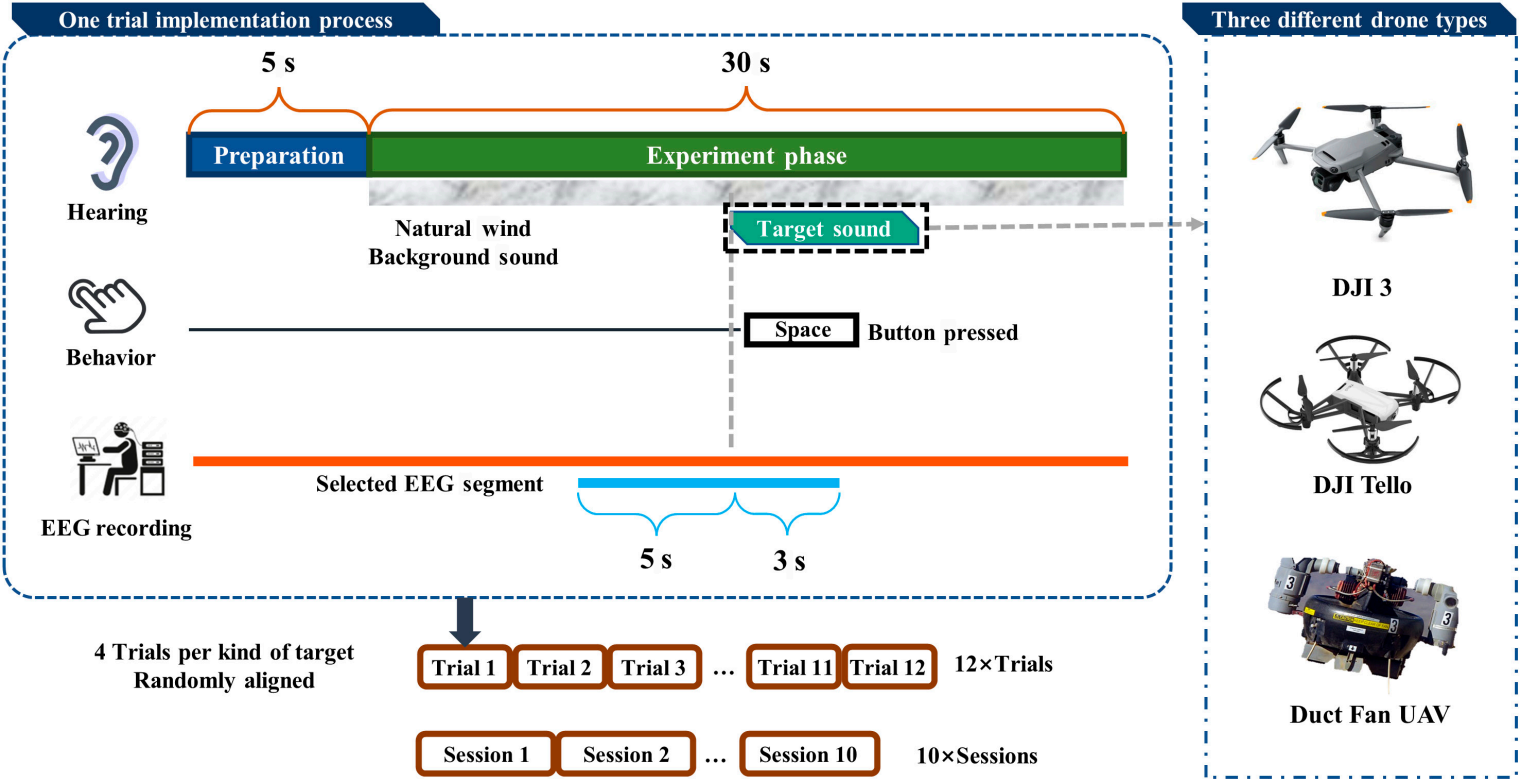
Illustration of the offline experimental protocol.

Eight participants (an average age of (23.8 ± 2.3) years, 5 males and 3 females) participated in the offline experiment, all right-handed and without prior BCI experience. All participants met strict inclusion criteria: no hearing-related diseases/disorders, and abstinence from alcohol, caffeine, or psychoactive substances for ≥24 h before experiments. This experiment passed the ethical review of the Beijing Institute of Technology (approval number: BIT-EC-H-2024150) and strictly complied with the Declaration of Helsinki (2013). The experimental paradigm is presented in Fig. 1.

During the experimental preparation phase, participants wore EEG acquisition caps and in-ear headphones. The experiments were divided into 10 sessions, each containing 12 trials. Each trial consisted of a preparation phase of 5 s and an experimental phase of 30 s. Participants were required to hear a segment of environmental noise (natural wind noise) containing a sound target, an unmanned aerial vehicle (UAV), through headphones. They were asked to gently press the space bar on their keyboard as soon as possible after they heard the sound target. To introduce spectral diversity and assess generalized auditory pattern recognition, 3 distinct drone types with markedly different acoustic characteristics were presented 4 times each per session (12 presentations in total) in a pseudo-random order.

Sixty channels of EEG signals were collected using NeuroScan SynAmps2 (NeuroScan, America), and the positions of the channels were determined by the international 10-20 system standard. The UAV sound samples in the experiment were recorded in an anechoic chamber environment. The natural wind recorded by DJI Mic (DJI, China) was used as the ambient noise sample, the sampling frequency was 44,100 Hz, and the ambient wind level was 6 to 8 during recording. The SNR of the sound targets used in the experiments was set to be low with respect to the environmental noise, which is −13.63 dB on average.

We time-locked the analysis to an 8-s EEG epoch (−5 to + 3 s relative to target onset), consistently applied across preprocessing, event-related potential (ERP) averaging, and model training. Preprocessing included baseline correction (first 1 s), artifact trial rejection, finite impulse response (FIR) bandpass filtering (1 to 49 Hz) and common average re-referencing. Eye and EMG artifacts were removed using independent component analysis (ICA). Components classified as “eye” or “muscle” with >80% confidence were rejected. More details about the experimental paradigm and EEG preprocessing can be found in Ref. [18].

### Neural signature analysis

To enhance our understanding of the neural representations underlying auditory target perception in complex acoustic scenes, and to provide anatomical guidance for the subsequent model design, we employed a source analysis approach to estimate the cortical sources of brain activity. Source analysis was performed by first dividing the brain volume into stereo grids (modeling forward conduction) and then solving for the firing activity of dipoles at each grid vertex (solving source spatial information in reverse). The symmetric boundary element method was used to establish the forward conduction model of EEG signals [22]. Standardized low resolution brain electromagnetic tomography was used to estimate dipole firing activity at mesh vertices [23]. We established regions of interest (ROIs) to further analyze activity in specific brain regions. The definition of each brain region in the Desikan–Killiany atlas was adopted, and the established cerebral cortex model was divided into 69 subparts, 14 of which were selected for study [24].

In this study, source analysis was implemented through Brain Storm [25]. The results of the source analysis were used to build the EEG decoding model. More details about the neural signature in the time and frequency domains of this study can be found in Ref. [18].

### Tri-SDANet

**Fig. 2.**
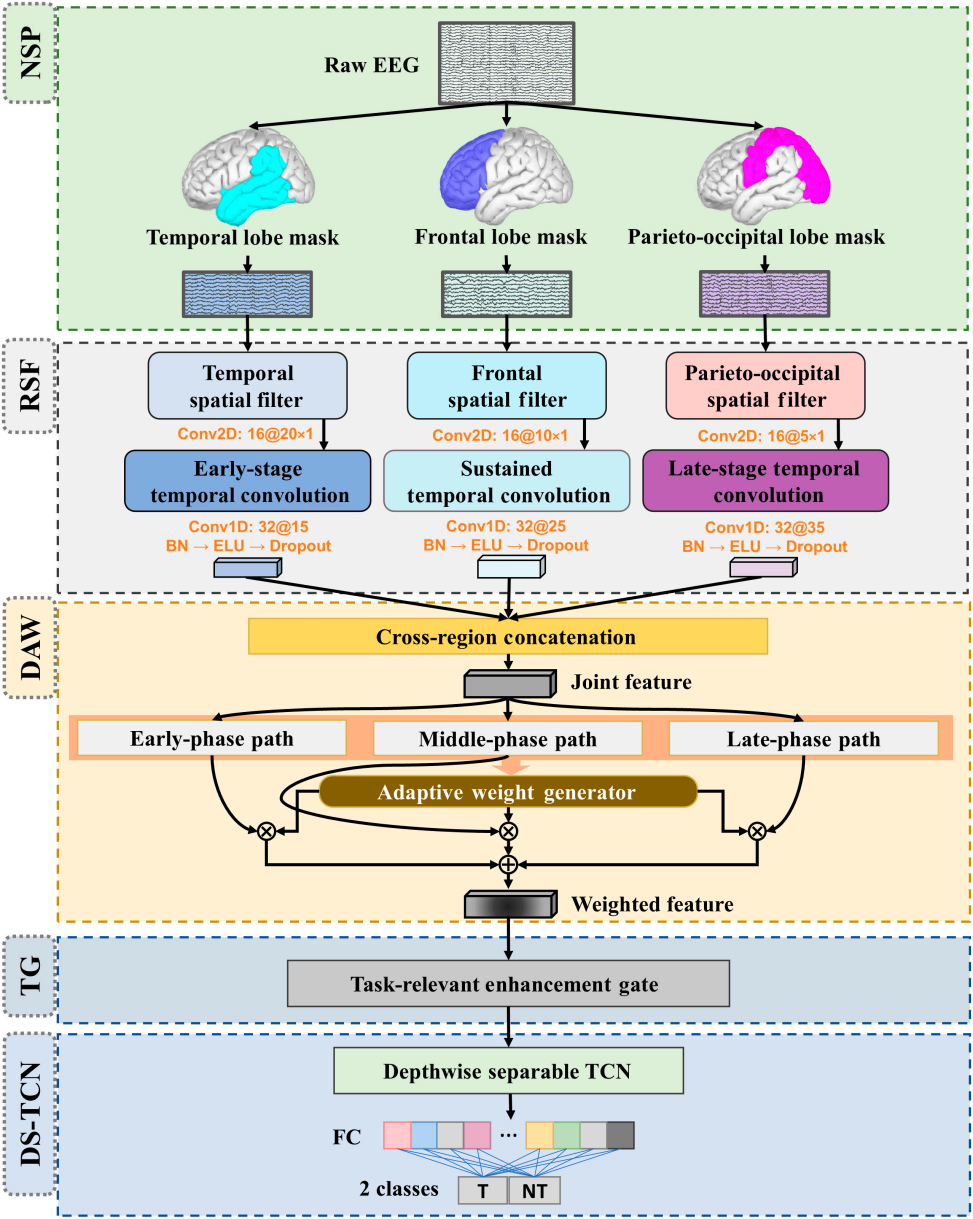
The structure diagram of Tri-SDANet. EEG signals are partitioned into temporal, frontal, and parieto-occipital regions, followed by region-specific spatiotemporal filtering, multi-scale temporal attention, temporal gating, and depthwise separable temporal convolution for auditory target decoding.

Based on the spatiotemporal activation characteristics of multiple brain regions induced by auditory target detection tasks, we constructed the Tri-SDANet. Although the architecture of Tri-SDANet is introduced first in this section for structural clarity, its module design was developed based on prior source-level EEG analysis. These neuroscientific findings are presented in the “Neural signature results” section and serve as the foundation for the biologically guided design choices implemented in Tri-SDANet. This network consists of 5 core modules: neuroanatomy-based spatial partitioning (NSP), region-specific spatiotemporal filtering (RSF), dynamic attention weighting (DAW), temporal gating mechanisms (TG), and depthwise separable temporal convolution (DS-TCN). The algorithm design closely aligns with neural representations, leveraging prior anatomical knowledge to enhance feature interpretability and conform to the spatial distribution characteristics of neural activity. The network input is multi-channel EEG signals, and the output is the probability distribution of task categories. The following content provides a detailed introduction to the algorithm. The relevant pseudo-code can be found in the Algorithms 1 to 3 in the Supplementary Materials. The schematic structure of Tri-SDANet is shown in Fig. 2.

#### Neuroanatomy-based spatial partitioning

This study follows the international 10-20 EEG electrode system standard to construct a channel-to-brain-region mapping matrix, dividing the 60-channel EEG signals into 3 functional modules: the temporal lobe, frontal lobe, and parieto-occipital lobe. The specific partitioning strategy is as follows:

• Temporal lobe region (temporal region): Composed of the T7–T8 horizontal axis (T3/T4, T5/T6), TP7–TP8, and extended electrodes, totaling 20 channels.
• Frontal lobe region (frontal region): Composed of the Fp1–Fp2 vertical axis (AF3/AF4, F3/F4, Fz), FC1–FC2, and adjacent electrodes, totaling 20 channels.
• Parieto-occipital lobe region (parieto-occipital region): Composed of the P3–P4 horizontal axis (PO3/PO4, O1/O2), CP1–CP2, and extended electrodes, totaling 20 channels.

Partitioned signals are denoted as $X_{k}\in {\mathbb{R}}^{C_{k}\times T}$ where $k\in \left\{T,\;F,\;P\right\}$ represents temporal/frontal/parieto-occipital regions. $C_{k}=20$ is channel count per region. T is temporal sampling points.

#### Region-specific spatiotemporal filtering

Three independent sets of spatial convolution kernels are used to process the electrode signals of the temporal, frontal, and parieto-occipital lobes, respectively. The wider temporal lobe filter preserves the complete spatial pattern of the primary auditory cortex, corresponding to its broader receptive field. The frontal lobe employs a medium-scale convolution kernel, reflecting its intermediate-range connectivity, while the downsampling in the parieto-occipital lobe corresponds to its dispersed spatial response characteristics. Specifically, the design of each filter is as follows:

• Temporal lobe filter: $W_{T}\in {\mathbb{R}}^{16\times 20\times 1}$, no padding
• Frontal lobe filter: $W_{F}\in {\mathbb{R}}^{16\times 10\times 1}$, edge padding of 4 units
• Parieto-occipital lobe filter: $W_{P}\in {\mathbb{R}}^{16\times 5\times 1}$, stride-2 downsampling

Fk=ELUWk·Xk,k∈TFP (1)

where ∗ denotes 2D spatial convolution and ELU is the exponential linear activation function.

To reflect the temporal characteristics of brain region activations during auditory target detection tasks and capture responses at different stages, we designed temporal convolutions of varying scales for each brain region’s signals. Specifically:

• Temporal lobe branch: 15-point convolution kernel (covering 150 ms).
• Frontal lobe branch: 25-point convolution kernel (covering 250 ms).
• Parieto-occipital lobe branch: 35-point convolution kernel (covering 350 ms).

Tk=Dropoutp=0.5ELUBNWkt·Fk (2)

where $W_{k}^{t}\in {\mathbb{R}}^{D\times S_{k}}$ is temporal kernel ($S_{k}$: kernel size); BN is batch normalization; ${\text{Dropout}}_{p=0.5}$ means dropout regularization (rate = 0.5).

#### Dynamic attention weighting

EEG signals contain different ERP components appearing in distinct time windows. The multi-scale attention mechanism captures features at early, mid, and late stages by using convolution kernels of varying scales and dynamically generating weights to fuse this information. This mechanism enables interpretable dynamic feature representation for EEG decoding through time-varying feature reorganization and adaptive receptive field adjustment. The mathematical description of the multi-scale temporal attention model is as follows:

Fconcat=TTTFTP (3)

A=∑s∈EMLαs·Ws·Fconcat (4)

where E/M/L correspond to 15/35/55-point convolution kernels, respectively, and the attention weights α are generated via softmax:

α=SoftmaxWa·FconcatEFconcatMFconcatL (5)

#### Temporal gating module

To enhance task-relevant neural marker features, we designed a temporal gating module in Tri-SDANet. Specifically, this module creates a mask with the same shape as the input, applying learnable gating parameters only to specific time windows and adjusting the activation intensity of these regions via a sigmoid function to amplify features at critical time points. The mathematical expression is:

G=σM⊙A (6)

where M is the temporal mask, σ is the sigmoid function, and ⊙ denotes the Hadamard product.

#### Depthwise separable temporal convolution

We designed a depthwise separable TCN using a depthwise convolution + pointwise convolution structure to simulate hierarchical information integration in the dorsal attention network (DAN) regions such as precentral gyrus (PreCG) and superior frontal gyrus (SFG):

Hd=ELUBNWd·G (7)

Hp=ELUBNWp·Hd (8)

where $W_{d}$ is the depthwise convolution kernel, and $W_{p}$ is the pointwise convolution kernel. Global average pooling is applied to obtain global features for each channel.

Zglobal=1T∑t=1THpt (9)

The TCN output is mapped to the number of categories via a fully connected layer.

h1=ELUWc1Zglobal+b1 (10)

y^=SoftmaxWc2h1+b2 (11)

#### Experimental setting of offline decoding

Based on the moment of target appearance, the [−3, −2] s EEG signals before the appearance of each trial target were used as the “nontarget sample”, and the [0,1] s EEG signals were used as the “target sample”. A common decoding model was trained for all subjects. We used 70% of all data for training, 10% for validation, and 20% for testing. Batch size was set to 8 in the model training process, a total of 200 epochs were trained, and Adams optimizer was used to adjust the weight parameters of each layer of the network.

### Automatic STD model

Given the research objective of achieving STD through human–machine collaboration, our study necessitates the incorporation of high-performance automated STD algorithms. We integrate 3 state-of-the-art detection models—GT-CNN [26], PaulNet [27], and Dual Branch CNN [28]—into our brain–machine fusion framework, where they serve as system components and comparative benchmarks. These algorithms were selected based on their proven efficacy, with GT-CNN originating from influential publications, while Dual Branch CNN and PaulNet represent an award-winning architecture from the DCASE Challenge series.

We first extracted the log-Mel spectrogram through the following computational process: A 1-s audio segment sampled at 44.1 kHz underwent spectral analysis using fast Fourier transform (FFT) with a window length of 2,048 samples and a 345-sample hop size. The resulting spectral information was subsequently converted to logarithmic Mel-scale representation through 128 Mel filter banks. This spectrogram matrix served as the input layer for our automated detection module.

For the automatic detection module, we maintained consistency with the BCI offline experiments by using the same training dataset containing only DJI 3, DJI Tello, and Duct Fan UAV sounds. This approach ensured fairness between the BCI and automatic detection models while allowing us to test the system’s generalization capability when encountering new UAV types. The UAV audio clips were randomly combined with ambient wind recordings (6 to 8 Beaufort scale wind captured via DJI Mic) to constitute the positive class, while unadulterated natural wind samples formed the negative class. Eighty percent of the dataset was used for the training, and 20% was used for the validation to test the model performance corresponding to the current iteration step. In the training process, Adams optimizer was used to calculate the parameters of each layer of the neural network model. The learning rate was 0.00001, the batch size was set to 64, and 300 epochs were iteratively trained. During the model training process, the current epoch’s model will be saved if the FAR of the validation set is below 10% and its recall is above 90%.

The output of the network is [$X_{1},X_{2}$]; if $X_{1}>X_{2}$, the sound sample is judged to be environmental noise class. If $X_{1}<X_{2}$, then the sound sample is judged as the target class. On this basis, this study used [$X_{1},X_{2}$] through softmax mapping, as shown in Eq. (11) to obtain $P_{1}$ and $P_{2}$, which are positive numbers greater than 0 and less than 1, and the sum is 1. Therefore, $\left[P_{1},\;P_{2}\right]$ was used as the probabilistic representation that the sound sample belongs to the environmental noise class and the target class, namely, the confidence degree.

P1=eX1eX1+eX2P2=eX2eX1+eX2 (12)

### Brain–machine hybrid intelligent STD system based on the confidence mechanism

**Fig. 3.**
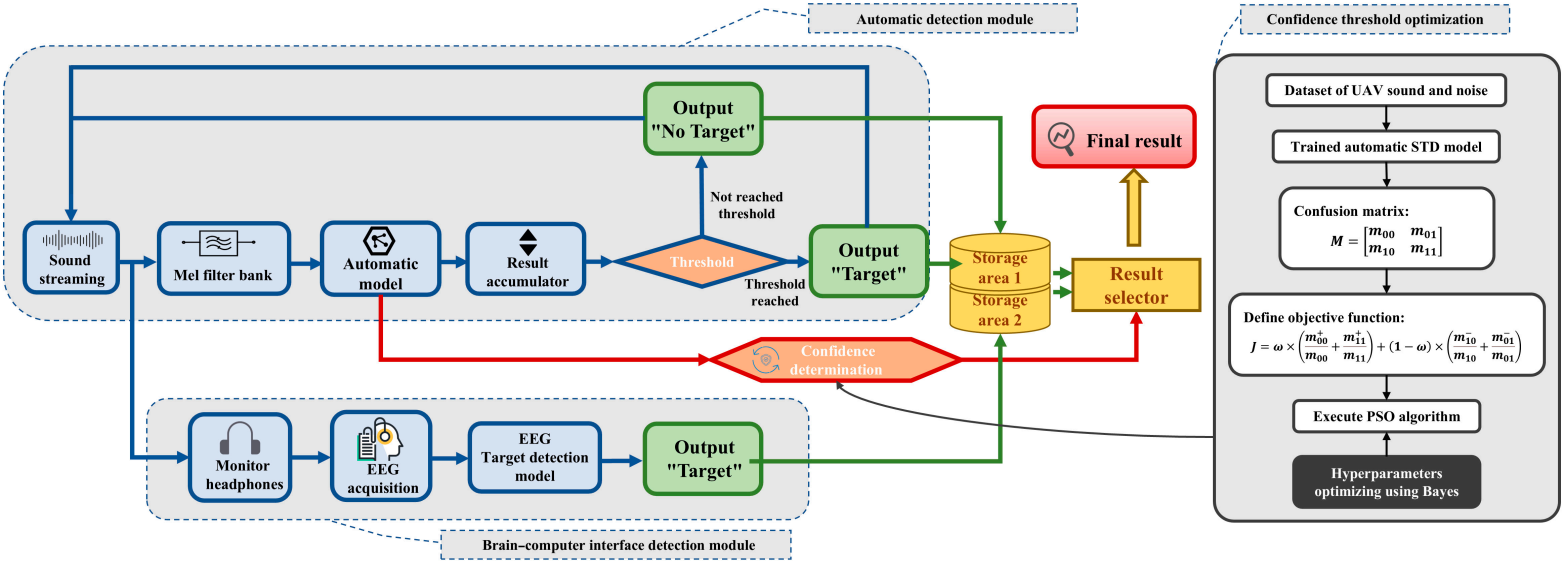
Architecture of brain–machine hybrid intelligent STD system based on confidence mechanism.

The architecture of the brain–machine hybrid intelligent STD system proposed in this paper is shown in Fig. 3. The overall STD system consists of 2 main parts: an automatic detection module and an auditory BCI-based module. The collected sound stream is input to both the automatic detection module and the monitor headphones. The automatic sound detection module extracts the Mel spectrum features of the sound segment, and input the features to the trained automatic STD model. The model outputs the discrimination result and discrimination confidence of the sound segment; if the discrimination result of the current segment is the target class, it adds one to the accumulator. Then, while waiting for the next sound segment, it is determined whether the value of the accumulator is greater than the detection threshold (the threshold was set to 3 in this paper). If the value is greater than the detection threshold, the output is “Target”; otherwise, the output is “No Target”. The output of the automatic detection module is stored in the memory area 1, and the discriminant confidence of the current time is output to the result selector module. While the automatic detection module processes and classifies the sound, the examiner manually judges the sound segments by listening to the headphones. The EEG acquisition system collects the EEG signals of the examiner and inputs them into the EEG decoding model proposed. Its output is stored in the memory area 2. Finally, according to the sound segment discrimination confidence output by the automatic sound detection module at the current time, the result selector selects one result from the memory regions 1 and 2 as the final target detection result.

The output of the brain–machine fusion system depends on the confidence in the automatic detection module; thus, the determination of the confidence interval threshold plays a crucial role in the performance of the final system. A good confidence interval, as an indicator to measure the “doubt degree” of the automatic detection system for the sound, should have the following characteristics:

1. The threshold should be as far as possible to classify the sound clips correctly judged by the automatic detection module into the “high confidence interval”, reduce the output of BCI commands, and reduce the FAR and human workload.
2. The threshold should classify the sound clips that the automatic detection module misjudges into the “low confidence” interval and then turn to the BCI to improve the robustness of the whole system against interference.

Based on the above rules, this study puts forward the evaluation criteria for the selection of the threshold of the confidence interval. Let the confusion matrix of the automatic detection module for a dataset be $M_{\text{Confusing}}$:

MConfusing=m00m01m10m11 (13)

In the above formula, $m_{\mathrm{ij}}$(i=0,1;j=0,1) respectively represents the number of samples with “true labeli, output label” for the corresponding case. The true label of the environmental noise is 0 and the true label of the sound target is 1.

The confidence interval evaluation function J is defined as:

J=ω·m00+m00+m11+m11+1−ω·m10−m10+m01−m01 (14)

In the above formula, $m_{\mathrm{ij}}^{+}$ and $m_{\mathrm{ij}}^{-}$ are respectively the number of “high confidence samples” and “low confidence samples” in the corresponding case according to the current confidence interval. $\frac{m_{00}^{+}}{m_{00}}+\frac{m_{11}^{+}}{m_{11}}$ is the proportion of sound clips that are correctly judged and classified as “high confidence interval” by the automatic detection module. According to characteristic #1, the larger the value, the better; $\frac{m_{10}^{-}}{m_{10}}+\frac{m_{01}^{-}}{m_{01}}$ is the proportion of samples that fall into the “low confidence interval” of the misjudged sound clips. According to characteristic #2, the larger the value, the better. Therefore, the larger the evaluation function J, the more the confidence interval can meet the requirements of the brain–machine fusion system proposed in this study. ω is used as the weight coefficient to adjust the influence of different terms, and the optimal ω value is obtained in the multi-objective optimization of the objective function J.

In this study, 100 sound clips of each of the 6 UAV types (DJI 3, DJI Tello, Duct Fan UAV, DJI Mini2, Red Eagle 5, and Hubsan H107D+) and 1,000 environmental noise sound clips that did not appear in the training set were selected to determine the best confidence threshold. Environmental noise samples and UAV sound samples were randomly combined as the dataset to determine the confidence threshold. The J values of $M_{\text{Confusing}}$ and different confidence thresholds of the automatic detection model were calculated on this dataset.

We optimized the objective function J through the particle swarm optimization (PSO) algorithm. Given the constrained 2-dimensional search space (confidence bound and weight parameter ω), the particle population was limited to 5 for computational efficiency. Bayesian optimization methodology was subsequently employed to determine the optimal hyperparameters, including inertia weight and acceleration constants $c_{1}$ and $c_{2}$. The optimization range of inertia weight was 0.4 to 1.4, and the optimization range of $c_{1}$ and $c_{2}$ was 0.2 to 2.0. More detailed pseudocode of the algorithm is provided in Algorithm 4 in the Supplementary Materials. In the operation of the proposed brain–machine hybrid STD system, the result selector selects the result output of memory region 2 (EEG decoding result) when $P_{1}$ is within the optimized low confidence interval, and the result output of memory region 1 (automatic detection result) in other cases.

### Streaming-mode detection experiment protocol

**Fig. 4.**
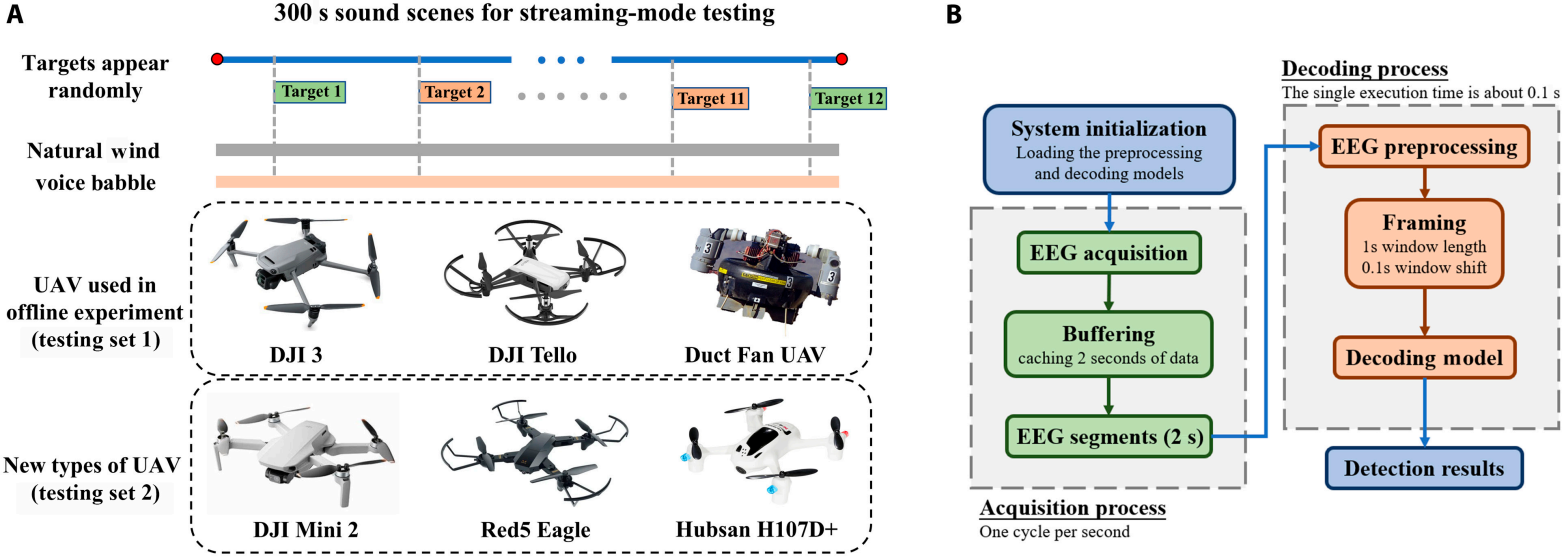
(A) Streaming-mode detection experimental protocol. (B) Working flowchart of auditory BCI streaming-mode detection experiment.

To evaluate the system’s performance under real-world application scenarios, we additionally designed a streaming-mode detection protocol based on a sequential decoding paradigm. This protocol continuously processes EEG signals and audio streams using a sliding window approach, thereby capturing key aspects of realistic auditory perception tasks, such as dynamic stimulus presentation, temporal continuity, and low-latency decision-making. This setup enables a rigorous assessment of the system’s temporal robustness and generalization ability in acoustically complex environments. The experimental procedure is illustrated in Fig. 4A. The experimental protocol comprised 20 auditory test scenarios, each lasting 300 s. To thoroughly assess the system’s robustness and generalization capability, we deliberately designed more complex and diverse acoustic environments. Each audio test scenario contained 6 types of UAV sounds (DJI 3, DJI Tello, Duct Fan UAV, DJI Mini2, Red Eagle 5, and Hubsan H107D+), with each UAV type appearing twice at different SNRs (−10 dB and 0 dB), resulting in 12 UAV sound events per scenario, and each UAV sound clip lasted 3 s. Among the 6 categories of UAVs, the 3 used in the offline experiment are classified as testing set 1, and the 3 newly added categories are classified as testing set 2 (Unseen targets). All UAV audio clips in the streaming-mode detection testing scenario remained nonoverlapping between the BCI offline experiments and the training sets of the automated detection module. The target sounds were presented in random order. To create more complex and varied acoustic environments than those in offline experiments, we used natural wind recordings (6 to 8 wind scale, captured by DJI Mic) mixed with voice babble from the noiseX-92 dataset as background noise [29].

Eight subjects (5 males and 3 females) aged 21 to 25 years (mean 23.5 ± 1.7 years) participated in the streaming-mode detection experiment. All auditory stimuli were precisely presented using the PsychoPy package [25].

The processing pipeline of the auditory BCI streaming-mode detection experiment, as illustrated in Fig. 4B, consists of 2 main modules (EEG signal acquisition and decoding) to simulate real-time processing in an online environment. A sliding window is employed to continuously load EEG signals with a window length of 2 s and a step size of 1 s in the acquisition process, ensuring temporal consistency with actual online experimental conditions. In each decoding cycle, the 2-s EEG signal is segmented into 11 overlapping 1-s segments using a sliding window of 1 s with a step size of 0.1 s. Each segment is independently fed into the EEG decoding model. If more than 5 out of the 11 segments are classified as the target class, the BCI detection system outputs a target detection decision.

The test audio segments are synchronously fed into the automatic detection algorithm with a window length of 1 s and a step size of 0.1 s. This algorithm outputs the discrimination confidence and result for each frame. The system simultaneously provides 3 types of outputs: BCI detection results, automatic detection algorithm results, and brain–computer fusion detection results based on the proposed confidence mechanism. To comprehensively evaluate the system’s performance, we simultaneously consider both recall and FAR as key metrics. A detection is considered a successful recall if the system outputs a “Target” within a 3-s window following the presence of a UAV. Conversely, any “Target” output occurring outside of this window is counted as a false alarm.

## Results

### Neural signature results

**Fig. 5.**
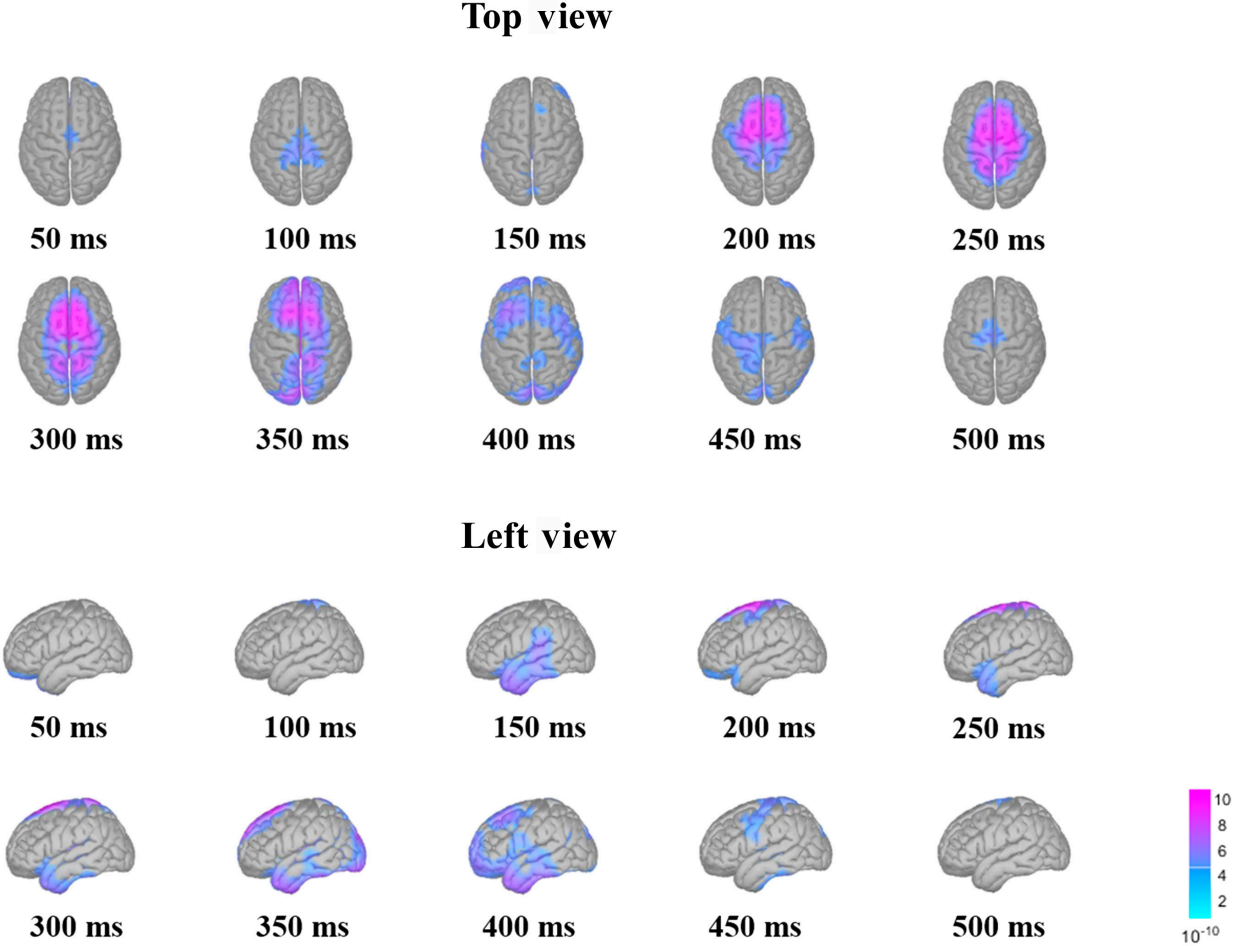
The grand-averaged activation pattern in the source space. The source imaging results are displayed from time lags 50 to 500 ms with the time interval of 50 ms. The source-space activity was imaged from 2 projection angles: top view and left view.

The estimated results of endogenous brain activity are shown in Fig. 5. According to Fig. 5, around 150 ms after the appearance of the sound target, the region near the temporal lobe of the brain showed significant activation. Subsequently, from 150 to 400 ms, sustained activation of the frontal and parietal lobes of the brain was observed, while transient activation of parts of the occipital lobe was observed from 350 to 450 ms.

The curves of different ROIs’ discharge intensity over time are listed in Fig. S1. After the sound target appeared, the right superior temporal gyrus (STG) and the adjacent supramarginal gyrus were activated first, and the activation onset was 134 and 132 ms, respectively. Subsequently, the postcentral gyrus and PreCG began to activate at 141 and 208 ms. The SFG and lateral occipital lobe (LO) began to activate around 300 ms after the occurrence of stimulation, and the first activation peak of the SFG was slightly earlier than the first activation peak of the LO.

In addition, 2 activation peaks appeared in the left STG, SFG, and PreCG. The second activation peak of the STG arrived earliest at 351 ms. PreCG was next at 477 ms. The second peak of activation in the left and right SFG appeared late.

**Fig. 6.**
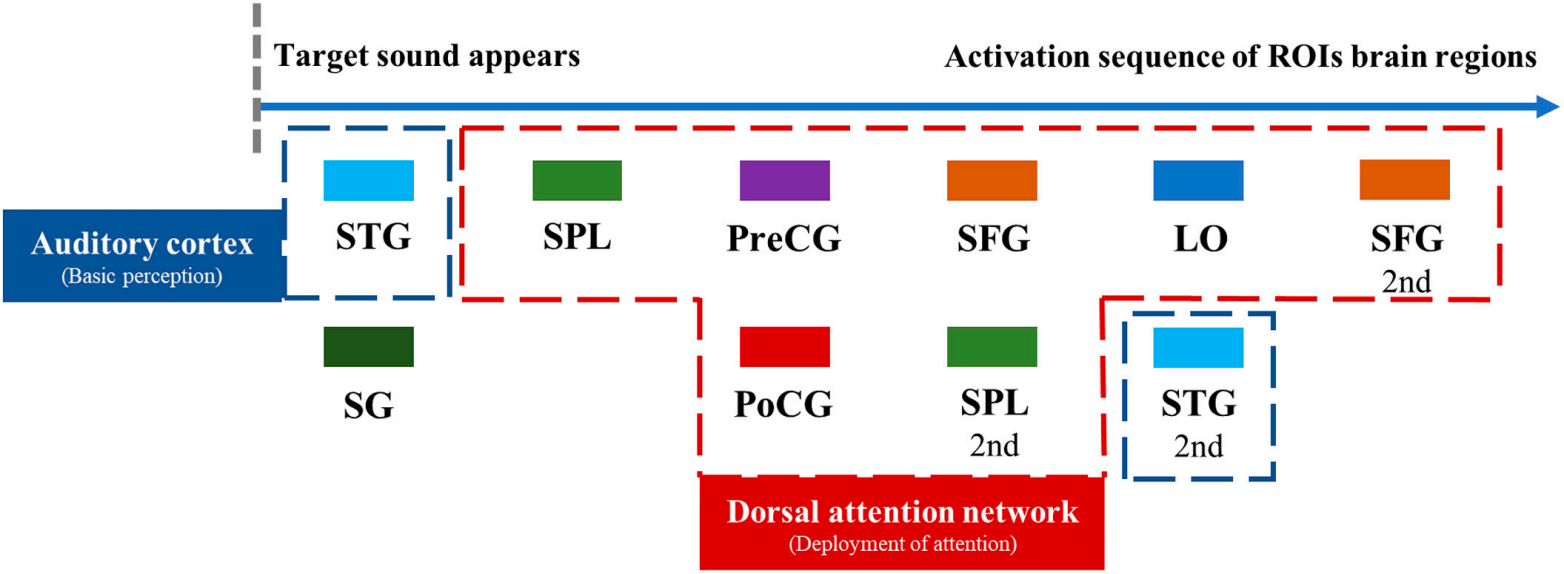
Activation sequence of the selected ROIs based on activation timing, showing initial STG activation; sequential engagement of PreCG, SFG, SPL, and LO; and subsequent reactivation of the STG.

According to the time when activation occurs, the activation sequence of each of the ROIs selected in this study can be plotted as Fig. 6.

Functionally, PreCG, SFG, superior parietal lobule (SPL), and LO are part of the DAN, which is primarily responsible for the “top-down” mechanism of attention orientation and is closely related to exogenous tasks [30]. This part of the brain was activated, suggesting in part that the participants’ attention was redeployed in response to the detection task and the presence of the sound target. After the auditory cortex, which is responsible for basic perception, extracts the features of the emerging sound (the first activation of the STG), the DAN undergoes a series of activation (information processing) that ultimately redeploys the auditory cortex’s attention to the auditory target (the second activation of the STG). From the relevant conclusions of neuroscience, this is basically consistent with the “bottom-up” perception process and the “top-down” regulation process of attention.

### Offline decoding performance

#### Compared with contrastive models

The proposed model was benchmarked against widely adopted contemporary methods, as presented in Table 1. Results were evaluated using the Wilcoxon signed-rank test (*P < 0.05; **P < 0.01; ***P < 0.001). Experimental results demonstrate that our method achieves significant advantages across 3 key metrics: accuracy (87.5% ± 3.9%), TPR (86.8% ± 5.5%), and FPR (8.3% ± 3.2%). Notably, compared to the closest-performing baseline, EEG-Conformer (85.3±5.1%), our method improves accuracy by 2.2 percentage points while exhibiting lower standard deviation (3.9% vs. 5.1%), indicating superior stability.

**Table 1.** Comparison of the proposed algorithm with other advanced algorithms (*P < 0.05, **P < 0.01, ***P < 0.001)

Model	Accuracy/%	TPR/%	FPR/%
Proposed	**87.5 ± 3.9**	**86.8 ± 5.5**	**8.3 ± 3.2**
EEG-Conformer [34]	85.3 ± 5.1**	84.2 ± 7.0*	**7.6 ± 3.5**
DeepConvNet [35]	84.1 ± 6.0***	**87.1 ± 6.9**	13.8 ± 6.1***
TFF-Former [36]	82.7 ± 7.2***	81.5 ± 8.3***	14.1 ± 6.8***
EEG-Net [37]	80.9 ± 6.5***	78.4 ± 7.8***	10.9 ± 5.3**
GRU [38]	79.2 ± 8.3***	83.7 ± 9.1*	21.4 ± 8.2***
LSTM [39]	76.5 ± 8.9***	75.8 ± 9.7***	23.6 ± 9.0***
Shallow-Convnet [40]	74.8 ± 9.1***	72.3 ± 10.3***	25.2 ± 9.5***
SVM [41]	72.1 ± 9.0***	70.2 ± 9.9***	28.5 ± 9.3***

From an architectural perspective, Transformer-based models such as EEG-Conformer and TFF-Former, while outperforming traditional deep learning approaches (e.g., EEG-Net and Shallow-ConvNet) in accuracy, still fall short of our method. This suggests that pure attention mechanisms may not sufficiently capture the spatiotemporal characteristics of EEG signals, highlighting the advantage of integrating neuroanatomical priors into the algorithm. Traditional machine learning methods (SVM) performed poorly, further validating the superiority of deep learning in EEG processing.

#### Ablation study

We conducted ablation experiments to perform an in-depth analysis of the 5 core modules in the Tri-SDANet model, aiming to reveal the contribution of each component to model performance. The ablation results are presented in Table 2. Experimental results demonstrate that the complete Tri-SDANet model achieved optimal performance, significantly outperforming all ablated variants in terms of accuracy (87.5% ± 3.9%) and F1 score (85.2% ± 4.1%). Notably, Model I (with NSP removed) showed the most substantial performance degradation, which fully confirms the importance of incorporating neuroanatomical prior knowledge into the model architecture.

**Table 2.** The results of the ablation study

#	NSP	RSF	DAW	TG	DS-TCN	Accuracy/%	F1 score
I	✗	✗	✓	✓	✓	75.2 ± 6.8 ^a^	72.4 ± 7.1 ^a^
II	✓	✗	✓	✓	✓	82.1 ± 4.3 ^a^	79.6 ± 4.7 ^a^
III	✓	✓	✗	✓	✓	83.7 ± 3.6 ^a^	81.5 ± 3.9 ^a^
IV	✓	✓	✓	✗	✓	85.1 ± 3.2 ^a^	83.7 ± 3.5 ^a^
V	✓	✓	✓	✓	✗	86.3 ± 3.1 ^a^	84.2 ± 3.3 ^a^
Tri-SDANet	✓	✓	✓	✓	✓	87.5 ± 3.9	85.2 ± 4.1

^a^ Indicates a decline in model performance.

In terms of individual module contributions, the removal of RSF led to a 5.4% accuracy drop, demonstrating its crucial role in multi-scale spatiotemporal feature extraction. The ablation of DAW and TG resulted in performance decreases of 3.8 and 2.4 percentage points, respectively, highlighting these modules’ key functions in feature selection and enhancement. While the removal of DS-TCN had a relatively minor impact, it still played a positive role. Moreover, this module’s smaller parameter count contributes significantly to computational efficiency optimization.

### Streaming-mode detection experiment results

#### Stand-alone BCI streaming-mode detection experiment results

**Fig. 7.**
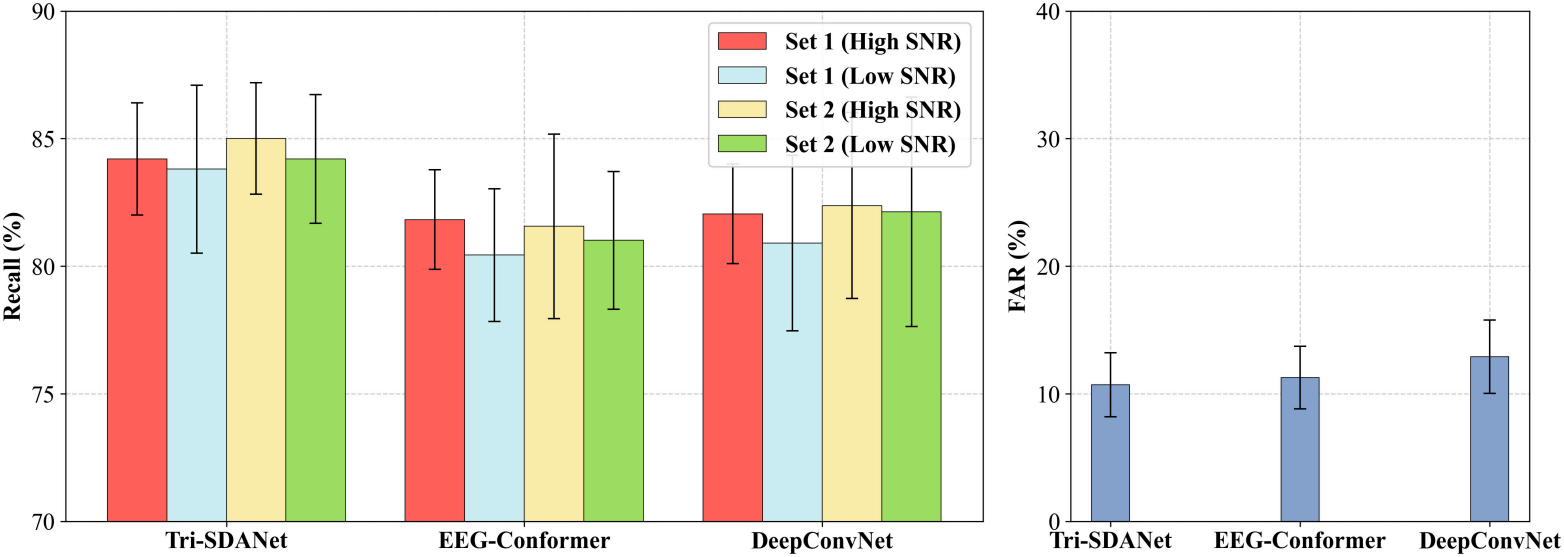
Stand-alone BCI streaming-mode detection testing performance. Recall across 4 testing sets and FAR of 3 algorithms. Tri-SDANet achieved the highest average recall with a lower FAR, while results remained stable across sets and SNRs, demonstrating the robustness and generalization of the BCI-based STD system.

This part conducted a systematic evaluation of the BCI output of the streaming-mode detection experiments of 8 subjects, with a focus on 2 core indicators: FAR and Recall. As shown in Fig. 7, the experimental results present different testing sets and SNRs, and conduct performance comparisons with the baseline algorithms (EEG-Conformer and DeepConvNet) that performed well in offline experiments. The complete individual-level data are detailed in Table S1.

Experimental data show that the Tri-SDANet model demonstrates advantages in the test, with an average Recall of 84.20% ± 2.53% and a FAR of 10.74% ± 2.51%. This result verified the feasibility of the STD system based on the auditory BCI and achieved the expected goal. However, due to the nonstationarity and low SNR of EEG signals, even though advanced algorithms have achieved performance improvements, the improvements are limited. The high FAR problem of BCI makes it impossible to be used as an independent STD system.

In terms of robustness and generalization, there was no statistically significant difference in the detection performance of BCI for the 2 testing sets (83.95% vs. 84.58%, Wilcoxon signed-rank, *P* = 0.56 > 0.05). It could maintain stable output under both high SNR and low SNR (84.58% vs. 83.94%, Wilcoxon signed-rank, *P* = 0.61 > 0.05). This result fully demonstrates that the STD method based on BCI has cross-SNR adaptability and multi-objective generalization ability, and has unique advantages in practical applications in complex acoustic environments.

#### Performance of automatic detection module

**Fig. 8.**
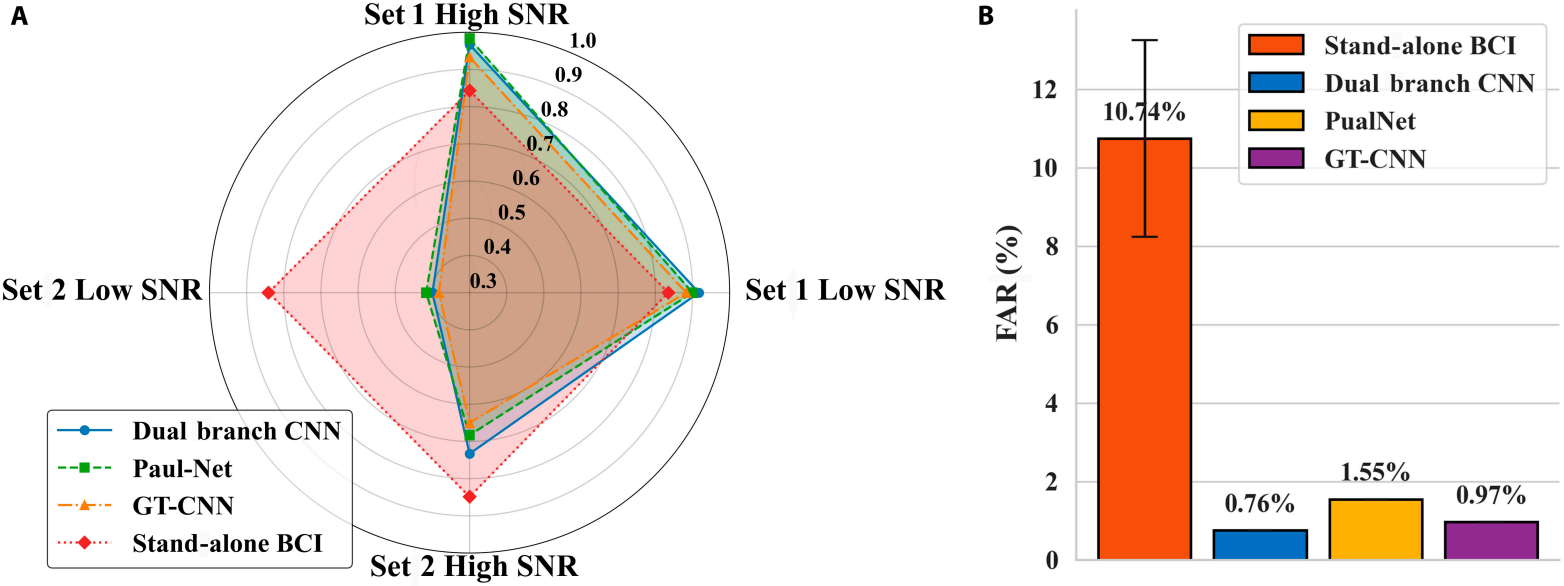
(A) Recall comparison of automatic detection modules (radar plot). (B) Comparison of FAR across various automatic detection modules (with reference to the BCI-based method).

Streaming-mode detection testing was performed for all 3 advanced automatic detection algorithms, and the performance of different modules is illustrated in the radar plot shown in Fig. 8. More detailed information is provided in the Supplementary Materials (Table S2), which presents the streaming-mode detection test recall results of the automatic detection modules, along with the corresponding confidence scores for successfully recalled instances. To better illustrate the respective advantages of the BCI-based approach and the automatic detection module, the radar chart also includes the results of the stand-alone BCI. It can be seen that the 3 automatic detection algorithms show similar results and rules, and the algorithm with the relatively best performance was Dual Branch CNN. The recall for testing set 1 was 94.17%, and it has high detection performance for both high SNR and low SNR targets. For testing set 2, which does not appear in the training set, the recall rate of the algorithm drops sharply, especially at low SNR, and the recall was only 40%. It is worth noting that the automatic detection module is able to maintain an extremely low FAR, which was 0.76% for the Dual Branch CNN.

In the confidence results from Table S6, the recognition confidence of the algorithm for testing set 1 was [0.36, 0.64]. However, the average confidence of testing set 2 in the successful detection process was [0.45, 0.55]. The results show that the automatic detection module has a good detection performance for the trained UAV types and is robust to SNR changes. However, for the new UAV that does not appear in the training set, the detection performance of the algorithm is significantly weakened, and the algorithm is more sensitive to the change of SNR. However, in the practical application of STD, new UAVs (rotor speed, blade profile, body size, etc.) often appear. In this case, the universality and robustness of the automatic detection module are low, which makes it unable to complete the target detection task independently.

#### Streaming-mode detection experimental performance of brain–machine fusion system

**Fig. 9.**
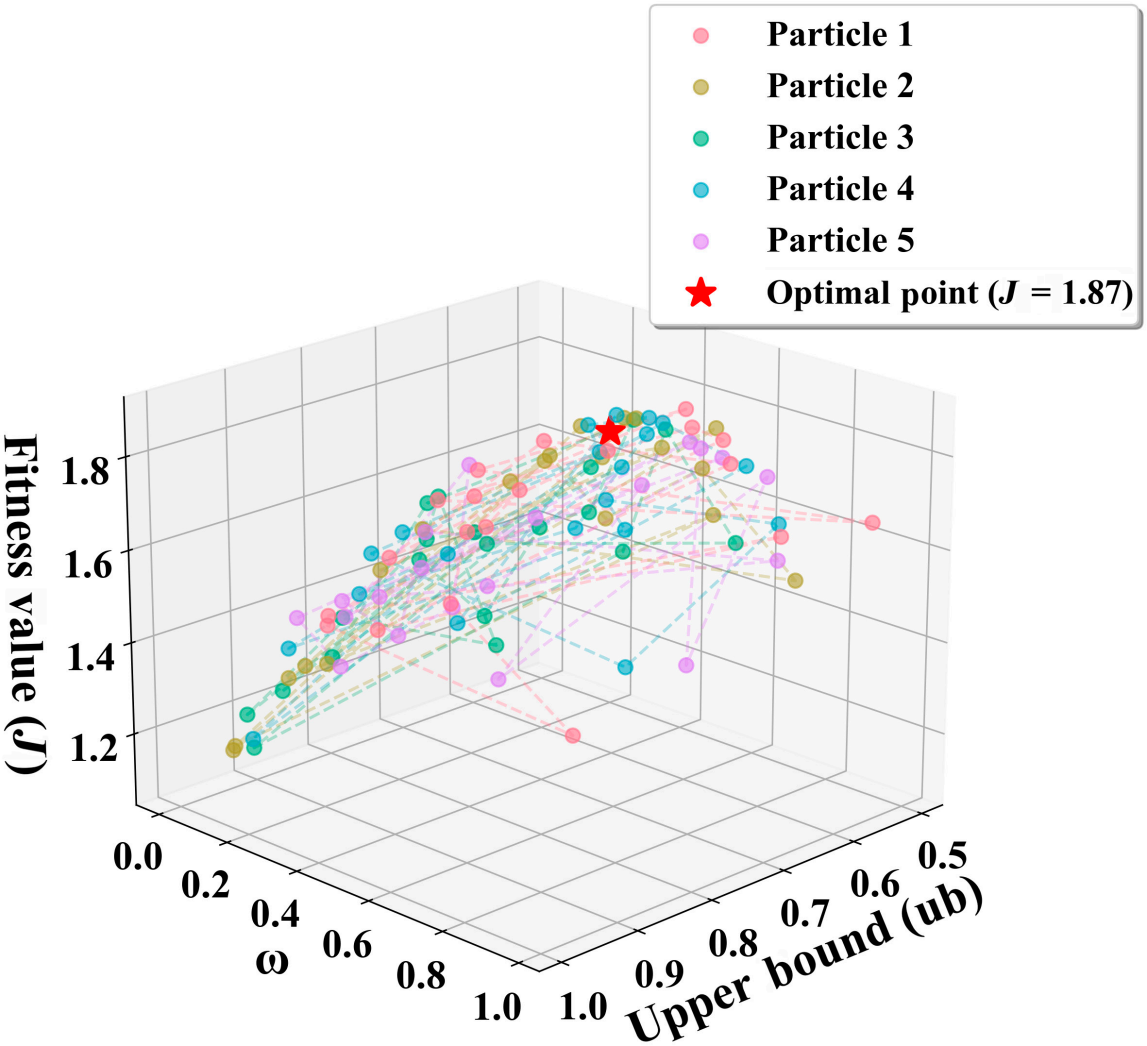
Particle movement paths in the search space during PSO optimization. Starting from a threshold of 0.5 for the environmental noise confidence $P_{1}$, particles converged to the optimal point, where the objective function achieved a maximum value of 1.87 under the selected parameters.

The BCI component and automated detection module within the brain–machine fusion system independently adopted their respective top-performing methods from streaming-mode detection testing evaluations—specifically, our proposed Tri-SDANet architecture and the established Dual Branch CNN framework. Based on the low FAR of the automatic detection module, we chose a threshold of 0.5 for the confidence $P_{1}$ of the environmental noise class as a starting point. The particle movement path in the search space during the implementation of the PSO algorithm is shown in Fig. 9. When the inertia weight was 0.729, $c_{1}$ and $c_{2}$ were 1.49, 1.65, $P_{1}$ interval was [0.5, 0.718], ω was 0.62, and the J value reached the maximum value of 1.87.

**Fig. 10.**
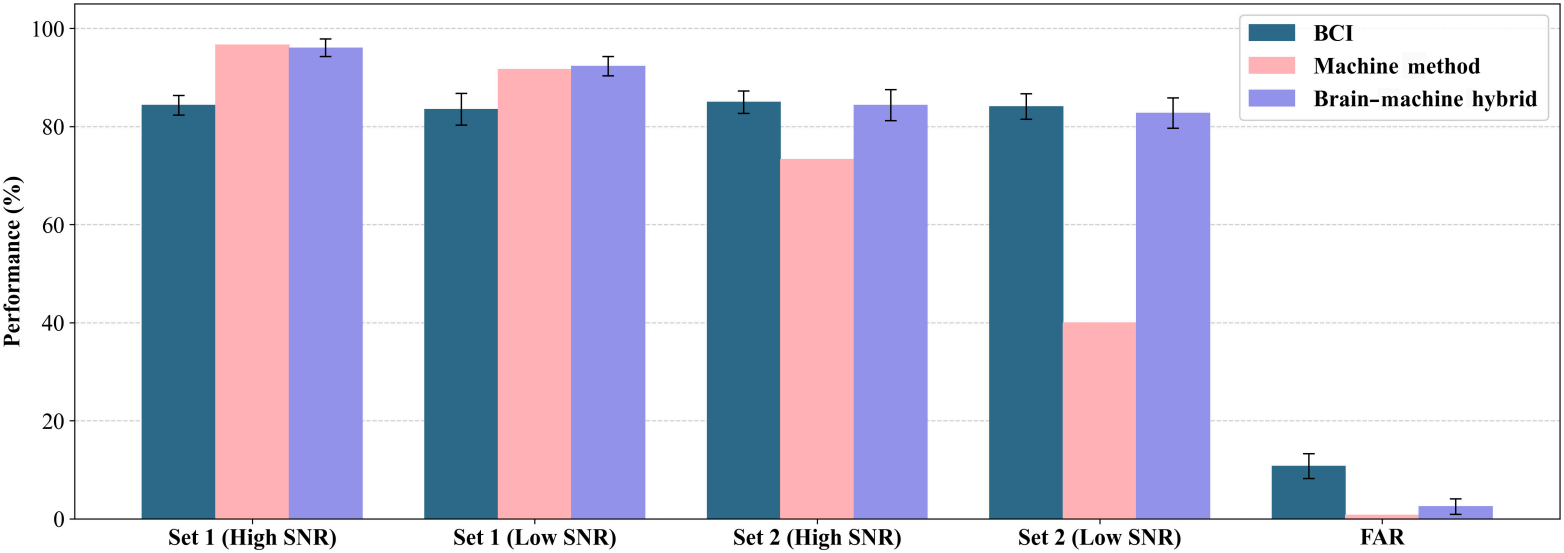
Comparison of streaming-mode detection experimental results of 3 methods (Recall and FAR). The hybrid approach improved recall (88.84%) over automatic detection and BCI, particularly for unseen targets (testing set 2), while reducing false alarms to 2.5%.

The final results of the streaming-mode detection experiment of the brain–machine fusion system are shown in Fig. 10. The proposed method makes full use of the advantages of both sides and obtains a reliable final performance. Compared with the automatic detection module, the detection recall was significantly improved, especially for new types of targets in testing set 2. Finally, the brain–machine hybrid system achieved an average recall of 88.84%. Recall was also improved compared to BCI (88.84% vs. 84.20%, Wilcoxon signed-rank, *P* = 0.0093 < 0.01), mainly in that the UAV type in testing set 1 was able to retain the high recall of the automatic detection module, and the FAR achieved a significant decrease relative to the BCI method, with the hybrid brain–machine system obtaining an average FAR of 2.5%.

In practical application scenarios, the monitor should select the participant with the best performance, so special attention should be paid to the performance of the optimal participant when evaluating the performance of the brain–machine fusion system. The performance of participant S2 achieved a high recall of 89.08% and a low FAR of 0.85%, demonstrating the feasibility and effectiveness of the proposed method in practice. The streaming-mode detection results of the brain–computer fusion system for all participants are shown in Table S3.

## Discussion and Conclusion

In the field of STD, especially in acoustic environments with low SNR and frequent emergence of new target types, designing STD systems with solid robustness and high generalization performance remains a challenge. Our proposed brain–machine hybrid intelligence approach addresses these problems by combining the complementary strengths of human and machine intelligence.

Our previous research has demonstrated the feasibility of applying an auditory BCI to target detection. Thanks to humans’ advanced perception of sound, the BCI can easily identify new types of sounds. This characteristic results in BCI having a significant advantage in dealing with the complex and changeable acoustic environment. It can make up for the lack of generalization performance of the automatic detection module. In this paper, our study begins by developing a neuroanatomically guided EEG decoding network, Tri-SDANet, which incorporates source analysis-derived anatomical priors into a spatial–temporal dual-attention architecture. By embedding these priors, Tri-SDANet enhances feature interpretability and task specificity, a critical innovation given that most existing EEG decoding methods are either general purpose or tailored to nonauditory tasks (e.g., RSVP [31] and motor imagery [32]). Compared to other advanced algorithms, Tri-SDANet achieves the highest overall balanced performance in complex auditory decoding tasks. Ablation studies further confirm that each model component contributes positively to decoding accuracy and reliability.

Building on the proposed EEG decoding network, we introduce a confidence-based fusion strategy that adaptively merges BCI-derived decisions with outputs from the automatic STD model. This confidence mechanism routes low-confidence acoustic detections to the BCI module for neural-based judgment, thereby mitigating false alarms and improving robustness. We additionally designed a streaming-mode detection experiment featuring increased acoustic complexity and a more diverse set of UAV target types, enabling a more rigorous evaluation of the system under conditions that better reflect real-world acoustic challenges, including broader noise conditions and greater target variability. Based on streaming-mode detection testing involving 8 human participants, the proposed hybrid system achieved an average recall of 88.84% and an average FAR of 2.5% under low-SNR and variable-target conditions, demonstrating its practical effectiveness and robustness in complex, time-sensitive acoustic environments. Moreover, for a streaming-mode detection system, it is important to consider latency and decision-making cycles, as they directly affect real-time responsiveness. In our system, EEG decoding for each 2-s segment took ~105 ms and the automatic detection module took ~31 ms, indicating that the combined processing time is sufficiently low for practical applications.

Of particular interest is the behavior of the system from the perspective of the confidence index. Although the algorithm exhibited limited robustness and generalization in identifying previously unseen target types (testing set 2), it did not completely misclassify these novel targets as background noise. This is evidenced by a significant difference in recognition confidence between the missed targets and pure environmental noise. Specifically, the confidence interval for missed targets was [0.67, 0.33], whereas for pure noise it was [0.85, 0.15]. In other words, the algorithm was able to perceive a distinction between novel targets and noise; however, the confidence level was insufficient to trigger a target classification decision. Notably, the average confidence scores for these missed targets fall within the low-confidence interval defined in this study. This subset of ambiguous cases, therefore, requires additional discrimination through human auditory intelligence. These findings further validate the effectiveness and necessity of the proposed confidence-driven brain–machine hybrid framework, as it enables targeted intervention in low-confidence situations to enhance detection performance.

Despite the remarkable progress in auditory BCIs in recent years, their application to STD is still relatively scarce. Although there are no directly related studies on auditory BCI-based STD for comparison, we can still draw on studies in the field of computer audition to assess the value and significance of this study. Similar to the research focus we set, Al-Emadi et al. [33] attempted to leverage diverse sound datasets to train a convolutional recurrent neural network (CRNN) to recognize UAV targets. To verify the generalization performance of the model, they further adopted a new brand of UAV sound samples for testing. However, the results showed that when tested with new UAV sound samples, the recall rate generally dropped, and even in some cases, the recall rate dropped sharply from 90% to 20%, which mainly depends on the similarity between sound samples of the training and test sets. Although increasing the number of training samples is recognized as the best way to improve the generalization ability of deep learning models, in practice, this method is complex, expensive, and difficult to implement. This highlights the unique value of our proposed brain–machine hybrid intelligence method and provides a new possibility to improve the robustness and generalization of STD systems.

However, some issues need to be considered in future work. First, integrating BCIs into real-time systems requires a focus on latency. This may require further optimization of algorithms and hardware devices to improve the response speed of the system. Second, the performance of the system can be affected by operator fatigue and training levels. Therefore, future research also needs to focus on reducing the burden on operators and improving the effectiveness of training to ensure system stability and reliability. Finally, extending this approach to a wider range of sound targets and acoustic environments requires further investigation and validation. In particular, future work should examine system performance across a broader spectrum of target categories and under more diverse noise conditions. This will likely necessitate the collection of additional datasets that capture such variability, thereby enabling a more comprehensive assessment of the system’s robustness and generalization.

In summary, this study contributes multiple significant advances to the field: (a) a pioneering neuro-acoustic fusion framework for robust STD, offering an application-ready solution for complex real-world acoustic scenarios; (b) targeted algorithm design that leverages source-derived anatomical priors to improve interpretability and task relevance; and (c) integration of a confidence-aware brain–machine fusion mechanism to complement and enhance automatic detection, yielding superior performance in challenging acoustic conditions. By combining the strengths of machines and humans, it is expected to develop systems that can adapt to new challenges and evolving threat environments, ensuring the effectiveness and reliability of STDs in the future.

## Data Availability

All data are available from the corresponding author upon reasonable request.

## Supplementary Materials

Supplementary 1 Algorithms 1 to 4 Fig. S1 Tables S1 to S3

## References

[1] Lopatka K, Czyzewski A. Acceleration of decision making in sound event recognition employing supercomputing cluster. Inf Sci. 2014;285:223–236.

[2] Waldekar S, Saha G. Analysis and classification of acoustic scenes with wavelet transform-based mel-scaled features. Multimed Tools Appl. 2020;79(11–12):7911–7926.

[3] Kong QQ, Xu Y, Wang WW, Plumbley MD. Sound event detection of weakly labelled data with CNN-transformer and automatic threshold optimization. IEEE ACM Trans Audio Speech Lan Process. 2020;28:2450–2460.

[4] Clavel C, Ehrette T, Richard G. Events detection for an audio-based surveillance system. In: *2005 IEEE International Conference on Multimedia and Expo 2005*. IEEE; 2005. p. 1306–1309.

[5] Papadimitriou I, Vafeiadis A, Lalas A, Votis K, Tzovaras D. Audio-based event detection at different SNR settings using two-dimensional spectrogram magnitude representations. Electronics. 2020;9(10):1593.

[6] Ren XD, Feng ZR, Lu HY, Zhou Q. Learning target template for acoustic event detection from low-SNR training data. IEEE Access. 2021;9:84490–84500.

[7] Li Y, Wu L. Detection of sound event under low SNR using multi-band power distribution. J Electron Info Technol. 2018;40(12):2905–2912.

[8] Li Y, Yin J. Sound event detection at low SNR based on multi-random forests. Acta Electron Sin. 2018;46(11):2705–2713.

[9] Li M, Wei R, Zhang Z, Zhang P, Xu G, Liao W. CVT-based asynchronous BCI for brain-controlled robot navigation. Cyborg Bionic Syst. 2023;4:0024. 37223547 10.34133/cbsystems.0024 PMC10202181

[10] Wang X, Guo S, Xu Z, Zhang Z, Sun Z, Xu Y. A robotic teleoperation system enhanced by augmented reality for natural human–robot interaction. Cyborg Bionic Syst. 2023;5:0098. 10.34133/cbsystems.0098 PMC11636702 39670176

[11] Wright BA, Zhang YX. A review of the generalization of auditory learning. Philosoph Trans Royal Soc B Biol Sci. 2009;364(1515):301–311. 10.1098/rstb.2008.0262 PMC2674478 18977731

[12] Barbour DL. Intensity-invariant coding in the auditory system. Neurosci Biobehav Rev. 2011;35(10):2064–2072. 21540053 10.1016/j.neubiorev.2011.04.009 PMC3165138

[13] Shamma S, Fritz J. Adaptive auditory computations. Curr Opin Neurobiol. 2014;25:164–168. 24525107 10.1016/j.conb.2014.01.011 PMC3981867

[14] Shi J, Xu X, Bi L, Feleke AG, Fei W. Low-quality video target detection based on EEG signal using eye movement alignment. Cyborg Bionic Syst. 2024;5:0125. 38966125 10.34133/cbsystems.0121 PMC11222288

[15] Lu ZH, Li Q, Gao N, Yang JJ. Time-varying networks of ERPs in P300-speller paradigms based on spatially and semantically congruent audiovisual bimodality. J Neural Eng. 2020;17(4): Article 046015. 32590363 10.1088/1741-2552/aba07f

[16] Kübler A, Furdea A, Haider S, Hammer EM, Nijboer F, Kotchoubey B. A brain-computer interface controlled auditory event-related potential (P300) spelling system for locked-in patients. Ann NY Acad Sci. 2009;1157:90–100. 19351359 10.1111/j.1749-6632.2008.04122.x

[17] Schreuder M, Blankertz B, Tangermann M. A new auditory multi-class brain-computer interface paradigm: Spatial hearing as an informative Cue. PLOS ONE. 2010;5(3): Article e9813. 20368976 10.1371/journal.pone.0009813 PMC2848564

[18] Wang RD, Liu Y, Shi JT, Peng BL, Fei WJ, Bi LZ. Sound target detection under noisy environment using brain-computer interface. IEEE Trans Neural Syst Rehabil Eng. 2023;31:229–237. 36331633 10.1109/TNSRE.2022.3219595

[19] Craik A, He YT, Contreras-Vidal JL. Deep learning for electroencephalogram (EEG) classification tasks: A review. J Neural Eng. 2019;16(3): Article 031001. 30808014 10.1088/1741-2552/ab0ab5

[20] Rahman MM, Sarkar AK, Hossain MA, Hossain MS, Islam MR, Hossain MB, Quinn JMW, Moni MA. Recognition of human emotions using EEG signals: A review. Comput Biol Med. 2021;136:104696. 34388471 10.1016/j.compbiomed.2021.104696

[21] Zhang X, Yao LN, Wang XZ, Monaghan J, McAlpine D, Zhang Y. A survey on deep learning-based non-invasive brain signals: Recent advances and new frontiers. J Neural Eng. 2021;18(3): Article 031002. 10.1088/1741-2552/abc902 33171452

[22] Clerc M, Gramfort A, Olivi E, Papadopoulo T. The symmetric BEM: Bringing in more variables for better accuracy. Berlin Heidelberg: Springer; 2010. p. 109–112.

[23] Pascual-Marqui RD. Standardized low-resolution brain electromagnetic tomography (sLORETA): Technical details. Methods Find Exp Clin Pharmacol. 2002;24:5–12. 12575463

[24] Alexander B, Loh WY, Matthews LG, Murray AL, Adamson C, Beare R, Chen J, Kelly CE, Anderson PJ, Doyle LW, et al. Desikan-Killiany-Tourville atlas compatible version of M-CRIB neonatal parcellated whole brain atlas: The M-CRIB 2.0. Front Neurosci. 2019;13:34. 30804737 10.3389/fnins.2019.00034 PMC6371012

[25] Tadel F, Baillet S, Mosher JC, Pantazis D, Leahy RM. Brainstorm: A user-friendly application for MEG/EEG analysis. Comput Intell Neurosci. 2011;2011:2011879716. 10.1155/2011/879716 PMC3090754 21584256

[26] Sen Gupta S, Hossain S, Kim KD. Recognize the surrounding: Development and evaluation of convolutional deep networks using gammatone spectrograms and raw audio signals. Expert Syst Appl. 2022;200:116998.

[27] Primus P. Reframing unsupervised machine condition monitoring as a supervised classification task with outlier-exposed classifiers. techreport. 2020.

[28] Jiang A, Zheng X, Qiu Y, Zhang W, Chen B, Fan P, Zhang W-Q, Lu C, Liu J. THUEE system for first-shot unsupervised anomalous sound detection. techreport. 2024.

[29] Varga A, Steeneken HJM. Assessment for automatic speech recognition: II. NOISEX-92: A database and an experiment to study the effect of additive noise on speech recognition systems. Speech Comm. 1993;12(3):247–251.

[30] Corbetta M, Shulman GL. Control of goal-directed and stimulus-driven attention in the brain. Nat Rev Neurosci. 2002;3(3):201–215. 11994752 10.1038/nrn755

[31] Zhou Y, Yang B, Wang C. Multiband task related components enhance rapid cognition decoding for both small and similar objects. Neural Netw. 2024;175: Article 106313. 38640695 10.1016/j.neunet.2024.106313

[32] Ke S, Yang B, Qin Y, Rong F, Zhang J, Zheng Y. FACT-net: A frequency adapter CNN with temporal-periodicity inception for fast and accurate MI-EEG decoding. IEEE Trans Neural Syst Rehabil Eng. 2024;32:4131–4142. 10.1109/TNSRE.2024.3499998 40030248

[33] Al-Emadi S, Al-Ali A, Al-Ali A. Audio-based drone detection and identification using deep learning techniques with dataset enhancement through generative adversarial networks. Sensors. 2021;21(15):4953. 34372189 10.3390/s21154953 PMC8348319

[34] Song YH, Zheng QQ, Liu BC, Gao XR. EEG conformer: Convolutional transformer for EEG decoding and visualization. IEEE Trans Neural Syst Rehabil Eng. 2023;31:710–719. 37015413 10.1109/TNSRE.2022.3230250

[35] Schirrmeister R, Gemein L, Eggensperger K, Hutter F, Ball T. Deep learning with convolutional neural networks for decoding and visualization of EEG pathology. In: *2017 IEEE Signal Processing in Medicine and Biology Symposium (SPMB)*. IEEE; 2017. p. 1–7.

[36] Li XJ, Wei W, Qiu S, He HG. TFF-Former: Temporal-frequency fusion transformer for zero-training decoding of two BCI tasks. Paper presented at: Proceedings of the 30th ACM International Conference on Multimedia, MM; 2022 Oct 10; Lisbon, Portugal.

[37] Lawhern VJ, Solon AJ, Waytowich NR, Gordon SM, Hung CP, Lance BJ. EEGNet: A compact convolutional neural network for EEG-based brain-computer interfaces. J Neural Eng. 2018;15(5): Article 056013. 29932424 10.1088/1741-2552/aace8c

[38] Cho K, Merrienboer B, Gulcehre C, Bougares F, Schwenk H, Bengio Y. Learning phrase representations using RNN encoder-decoder for statistical machine translation. In: *Proceedings of the 2014 Conference on Empirical Methods in Natural Language Processing (EMNLP)*. Doha (Qatar): 2014. p. 1724–1734

[39] Hochreiter S, Schmidhuber J. Long short-term memory. Neural Comput. 1997;9(8):1735–1780. 9377276 10.1162/neco.1997.9.8.1735

[40] Islam MR, Massicotte D, Nougarou F, Massicotte P, Zhu WP. S-Convnet: A shallow convolutional neural network architecture for neuromuscular activity recognition using instantaneous high-density surface EMG images. In: *2020 42nd Annual International Conference of the IEEE Engineering in Medicine & Biology Society (EMBC)*. IEEE; 2020. p. 744–749. 10.1109/EMBC44109.2020.9175266 33018094

[41] Burges CJC. A tutorial on support vector machines for pattern recognition. Data Min Knowl Disc. 1998;2(2):121–167.

