# Supplementary material for: Neuroanatomy-Informed Brain–Machine Hybrid Intelligence for Robust Acoustic Target Detection
Source: Cyborg Bionic Syst. 2025 Oct 17;6:0438. doi: 10.34133/cbsystems.0438 (PMC12531490; doi:10.34133/cbsystems.0438)
Supplement: Supplementary 1 — Algorithms 1 to 4 Fig. S1 Tables S1 to S3 [file cbsystems.0438.f1.docx]

**Supplementary Materials**


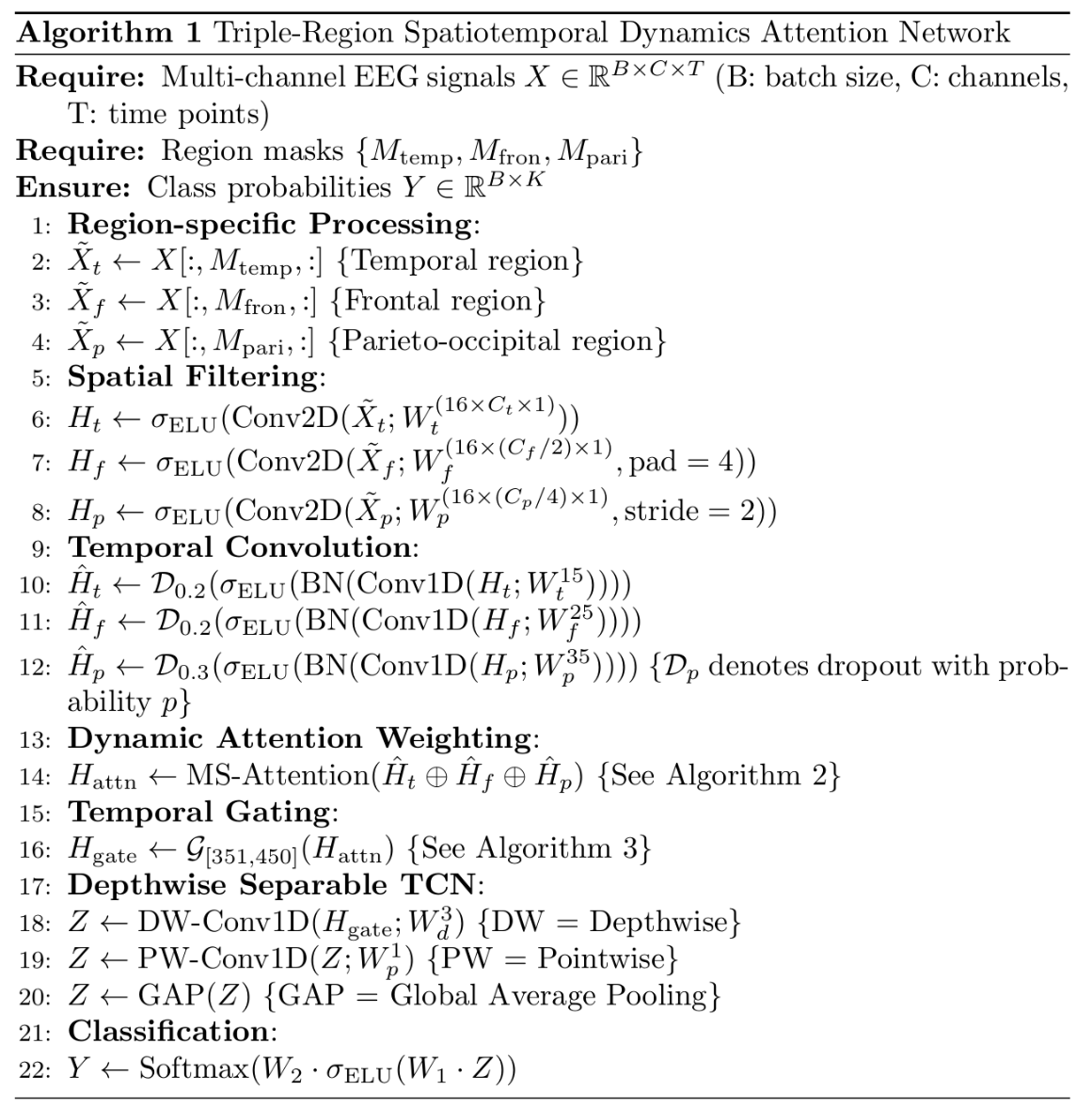


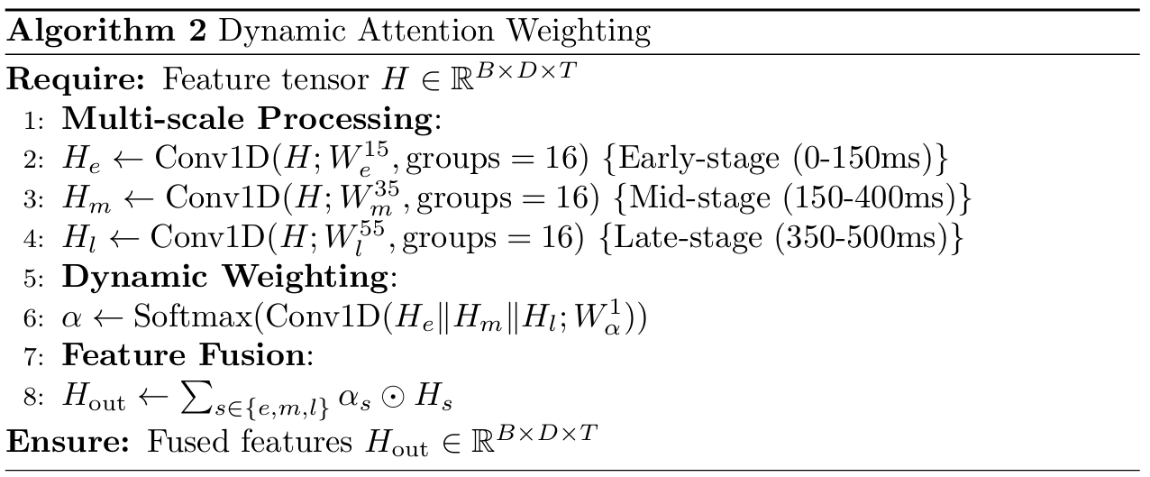


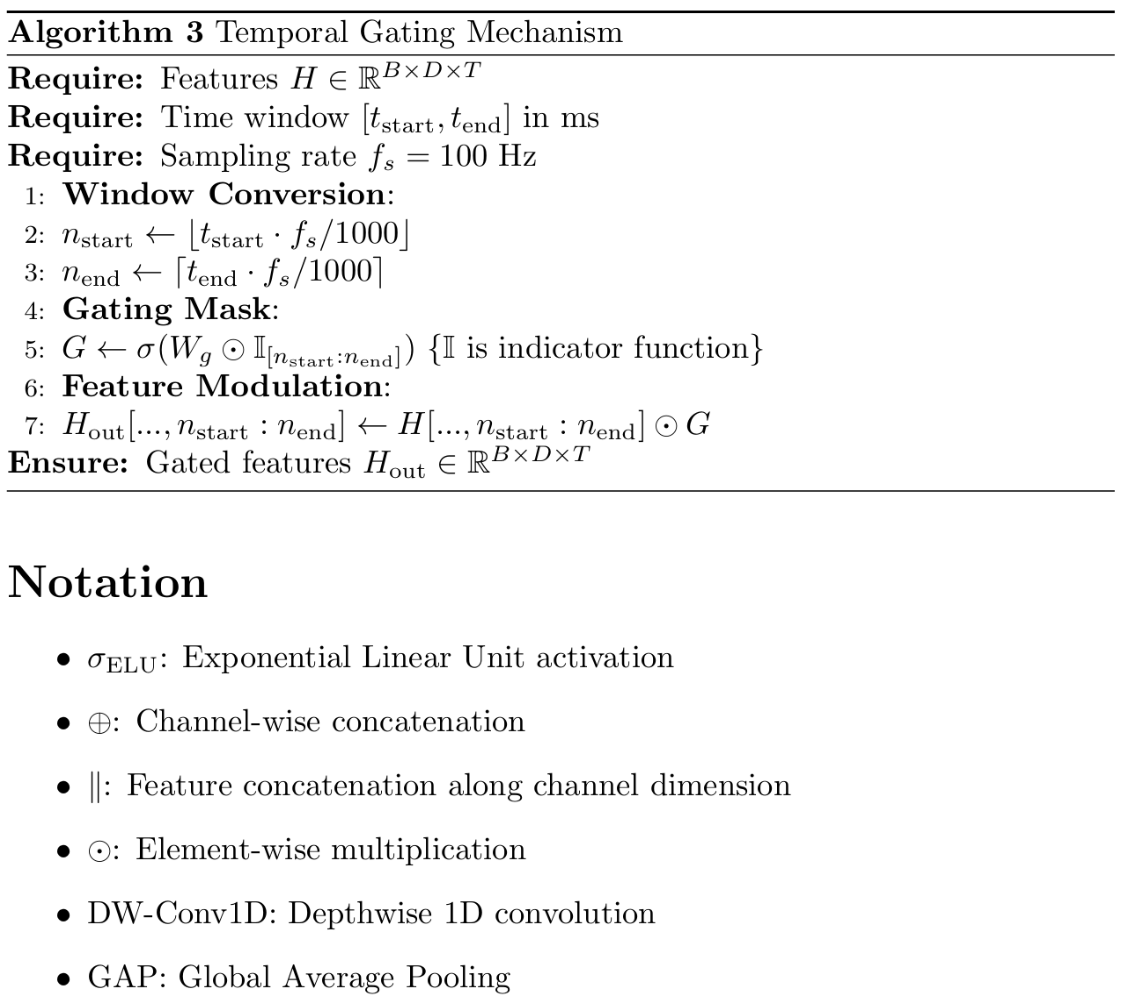


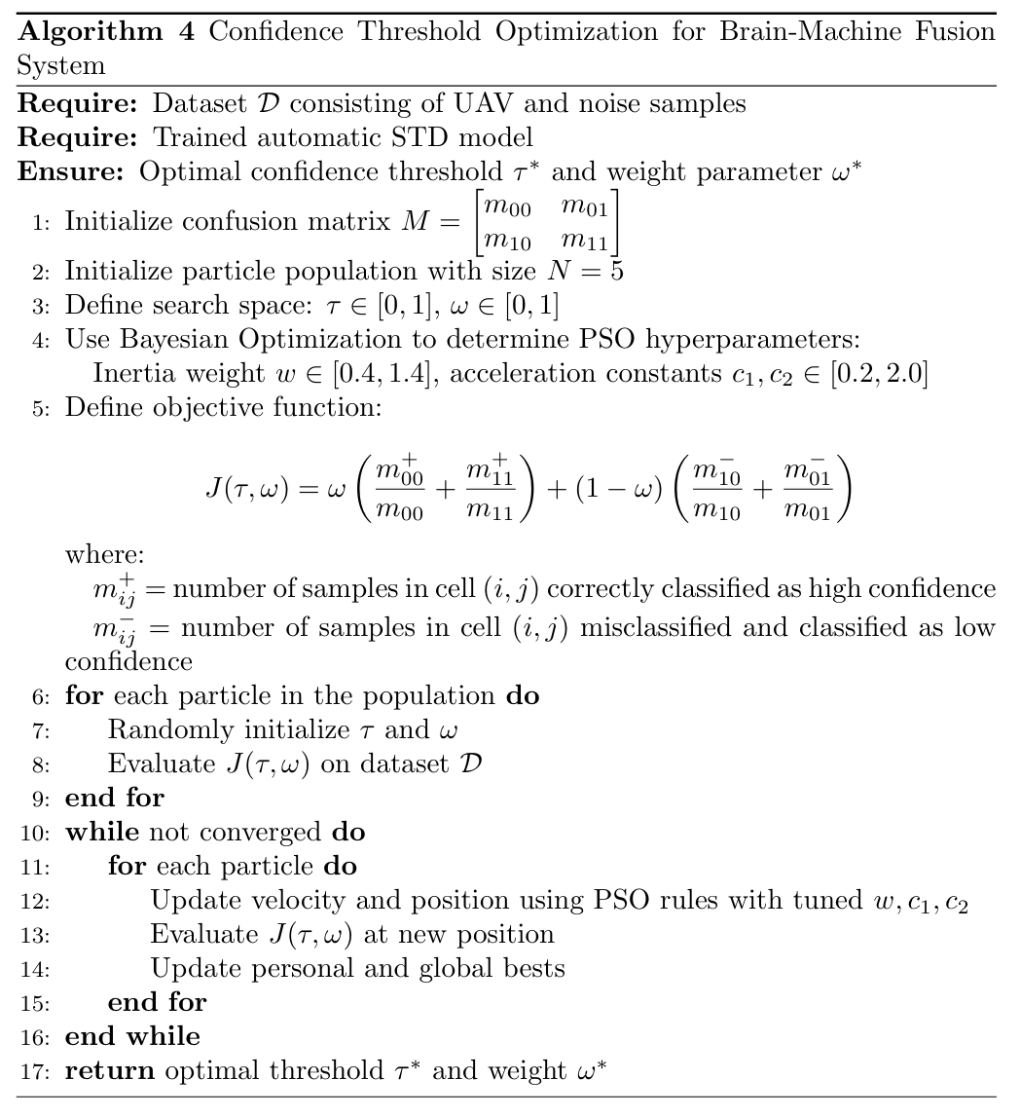


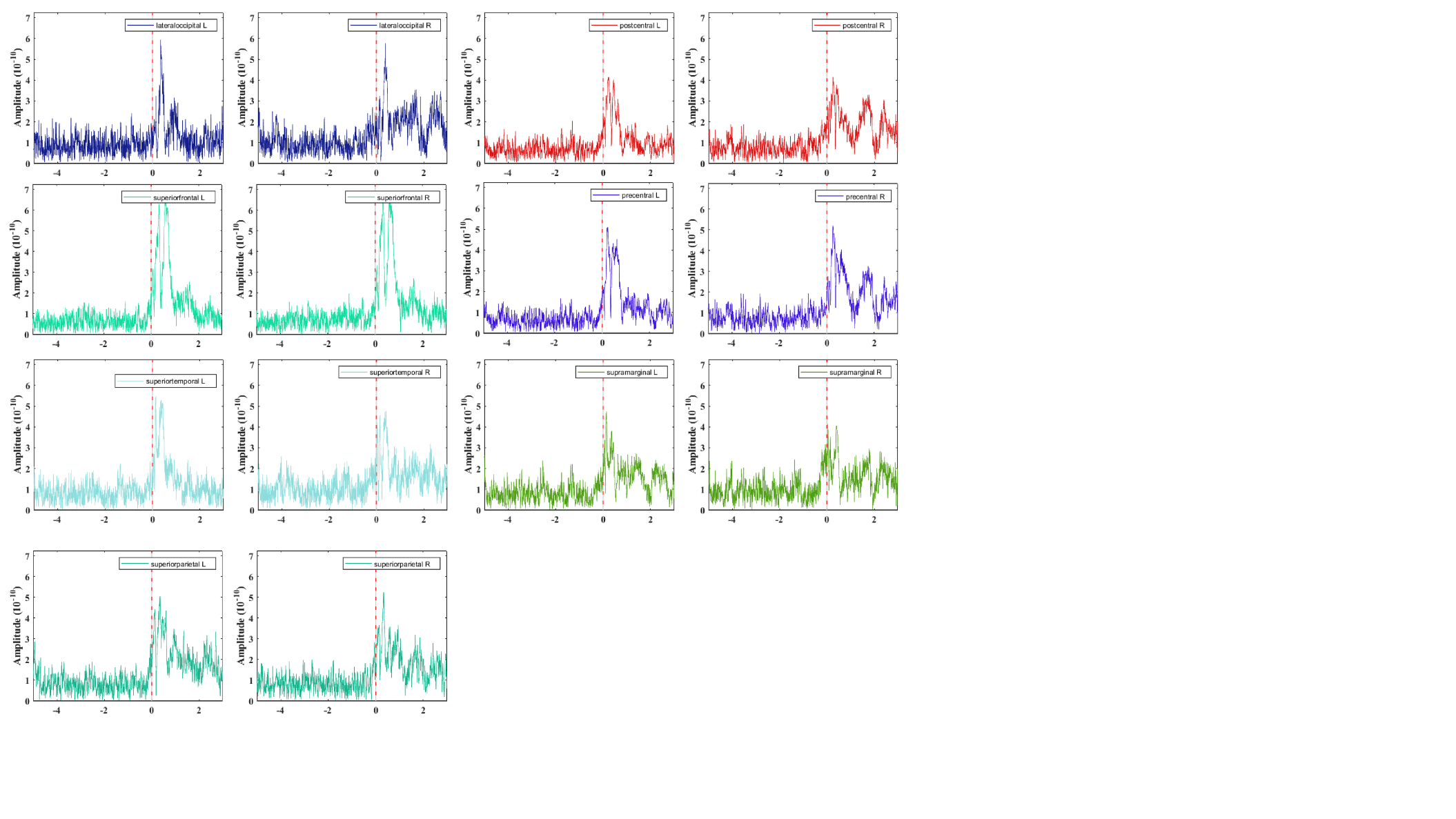


Fig. S1. The curves of 14 different ROIs discharge intensity over time.

TABLE S1

BCI detection performance of all participants in the streaming-mode detection experiment (%)

| Subject | Set1  (High SNR) | Set1  (Low SNR) | Set2  (High SNR) | Set2  (Low SNR) | FAR |
| --- | --- | --- | --- | --- | --- |
| 1 | 80.0 | 81.7 | 83.3 | 85.0 | 12.4 |
| 2 | 85.0 | 88.3 | 86.7 | 83.3 | 8.9 |
| 3 | 83.3 | 80.0 | 85.0 | 83.3 | 13.1 |
| 4 | 83.3 | 85.0 | 83.3 | 88.3 | 9.7 |
| 5 | 86.7 | 83.3 | 85.0 | 80.0 | 6.8 |
| 6 | 86.7 | 88.3 | 86.7 | 83.3 | 11.5 |
| 7 | 83.3 | 83.3 | 81.7 | 86.7 | 14.3 |
| 8 | 85.0 | 80.0 | 88.3 | 83.3 | 9.2 |
| ​Mean​ | ​84.2​ | ​83.8​ | ​85.0​ | ​84.2​ | 10.7 |
| ​​±Std​ | ​2.2​ | ​3.3​ | ​2.2​ | ​2.5 | 2.5 |

TABLE S2

Recall and confidence results of automatic detection module

|  |  | | All Samples | High SNR | Low SNR |  |
| --- | --- | --- | --- | --- | --- | --- |
| Dual Branch CNN | Testing Set 1 | Recall | 94.17% | 96.67% | 91.67% |  |
|  |  | Confidence | [0.36,0.64] | [0.33,0.67] | [0.38,0.62] |  |
|  |  |  |  |  |  |  |
|  | Testing Set 2 | Recall | 56.67% | 73.33% | 40.00% |  |
|  |  | Confidence | [0.44,0.56] | [0.42,0.58] | [0.46,0.54] |  |
|  |  |  |  |  |  |  |
| Paul-Net | Testing Set 1 | Recall | 94.17% | 98.33% | 90% |  |
|  |  | Confidence | [0.38,0.62] | [0.37,0.63] | [0.40,0.60] |  |
|  |  |  |  |  |  |  |
|  | Testing Set 2 | Recall | 55% | 68.33% | 41.67% |  |
|  |  | Confidence | [0.45,0.55] | [0.43,0.57] | [0.47,0.53] |  |
|  |  |  |  |  |  |  |
| GT-CNN | Testing Set 1 | Recall | 90.83% | 93.33% | 88.33% |  |
|  |  | Confidence | [0.40,0.60] | [0.39,0.61] | [0.40,0.60] |  |
|  |  |  |  |  |  |  |
|  | Testing Set 2 | Recall | 51.67% | 65% | 38.33% |  |
|  |  | Confidence | [0.47,0.53] | [0.46,0.54] | [0.48,0.52] |  |
|  |  |  |  |  |  |  |

TABLE S3

Overall performance of brain-machine fusion system (%)

| Subject | Set1  (High SNR) | Set1  (Low SNR) | Set2  (High SNR) | Set2  (Low SNR) | FAR |
| --- | --- | --- | --- | --- | --- |
| 1 | 95 | 91.67 | 81.67 | 83.33 | 2.65 |
| 2 | 95 | 93.33 | 86.33 | 81.67 | 0.85 |
| 3 | 96.67 | 88.33 | 85 | 78.33 | 3.98 |
| 4 | 96.67 | 95 | 78.33 | 85 | 1.67 |
| 5 | 98.3 | 93.33 | 85 | 81.67 | 0.92 |
| 6 | 95 | 91.67 | 86.67 | 80 | 1.88 |
| 7 | 93.33 | 93.33 | 83.33 | 88.33 | 5.50 |
| 8 | 98.33 | 91.67 | 88.33 | 83.33 | 2.57 |
| mean | 96.038 | 92.291 | 84.333 | 82.708 | 0.025 |
| ±std | 1.7621 | 1.9793 | 3.1724 | 3.0773 | 0.0158 |
